# Sensitivity of Diffusion MRI to White Matter Pathology: Influence of Diffusion Protocol, Magnetic Field Strength, and Processing Pipeline in Systemic Lupus Erythematosus

**DOI:** 10.3389/fneur.2022.837385

**Published:** 2022-04-26

**Authors:** Evgenios N. Kornaropoulos, Stefan Winzeck, Theodor Rumetshofer, Anna Wikstrom, Linda Knutsson, Marta M. Correia, Pia C. Sundgren, Markus Nilsson

**Affiliations:** ^1^Clinical Sciences, Diagnostic Radiology, Lund University, Lund, Sweden; ^2^Division of Anaesthesia, University of Cambridge, Cambridge, United Kingdom; ^3^BioMedIA Group, Department of Computing, Imperial College London, London, United Kingdom; ^4^Department of Medical Radiation Physics, Lund University, Lund, Sweden; ^5^Russell H. Morgan Department of Radiology and Radiological Science, Johns Hopkins University, School of Medicine, Baltimore, MD, United States; ^6^F.M. Kirby Research Center, Kennedy Krieger Institute, Baltimore, MD, United States; ^7^MRC Cognition and Brain Sciences Unit, University of Cambridge, Cambridge, United Kingdom; ^8^Lund University BioImaging Center, Lund University, Lund, Sweden; ^9^Department of Medical Imaging and Physiology, Skåne University Hospital, Lund, Sweden

**Keywords:** diffusion MRI, DTI, DKI, ROI-based analysis, ultra-high magnetic field strength (7T), diffusion processing, white matter fiber-tracts, effect sizes

## Abstract

There are many ways to acquire and process diffusion MRI (dMRI) data for group studies, but it is unknown which maximizes the sensitivity to white matter (WM) pathology. Inspired by this question, we analyzed data acquired for diffusion tensor imaging (DTI) and diffusion kurtosis imaging (DKI) at 3T (3T-DTI and 3T-DKI) and DTI at 7T in patients with systemic lupus erythematosus (SLE) and healthy controls (HC). Parameter estimates in 72 WM tracts were obtained using TractSeg. The impact on the sensitivity to WM pathology was evaluated for the diffusion protocol, the magnetic field strength, and the processing pipeline. Sensitivity was quantified in terms of Cohen's *d* for group comparison. Results showed that the choice of diffusion protocol had the largest impact on the effect size. The effect size in fractional anisotropy (FA) across all WM tracts was 0.26 higher when derived by DTI than by DKI and 0.20 higher in 3T compared with 7T. The difference due to the diffusion protocol was larger than the difference due to magnetic field strength for the majority of diffusion parameters. In contrast, the difference between including or excluding different processing steps was near negligible, except for the correction of distortions from eddy currents and motion which had a clearly positive impact. For example, effect sizes increased on average by 0.07 by including motion and eddy correction for FA derived from 3T-DTI. Effect sizes were slightly reduced by the incorporation of denoising and Gibbs-ringing removal (on average by 0.011 and 0.005, respectively). Smoothing prior to diffusion model fitting generally reduced effect sizes. In summary, 3T-DTI in combination with eddy current and motion correction yielded the highest sensitivity to WM pathology in patients with SLE. However, our results also indicated that the 3T-DKI and 7T-DTI protocols used here may be adjusted to increase effect sizes.

## 1. Introduction

Diffusion MRI (dMRI) can be used to characterize the microstructure of white matter (WM) fiber tracts by parameters obtained with for example diffusion tensor imaging (DTI). Examples of such parameters include the mean, axial, and radial diffusivity (MD, AD, and RD, respectively) and the fractional anisotropy (FA). Changes in these parameters have been detected in numerous conditions, including aging (1), traumatic brain injury (TBI) (2, 3), schizophrenia (4, 5), Parkinson's disease (6, 7), multiple sclerosis (MS) (8), and systemic lupus erythematosus (SLE) (9–12), (13–15). Diffusion kurtosis imaging (DKI) is an extension to DTI that provides information complementary to DTI (16–18), but requires a more comprehensive acquisition protocol and, thus, longer scan times. Whether to accept the longer scan times of a DKI protocol or to opt for a shorter DTI protocol is just one of the many questions scientists face when designing a dMRI protocol. Other questions may be what magnetic field strength to use, as it can also influence the outcome of a study (19). In addition, there are many questions concerning the choice of the processing pipeline, which can also impact the sensitivity of dMRI to pathology (20). An evaluation of all these aspects would enable a more informed choice of methods. Here, we evaluated three aspects: the diffusion protocol (DTI vs. DKI), the magnetic field strength (3T vs. 7T), and the processing pipeline (seven different options). The evaluation was based on a groupwise comparison of dMRI data from patients with SLE. This is a disease with a broad variety of symptoms of both a neurologic and psychiatric nature (21). Previous studies have reported reduced FA in the corpus callosum and a wide range of association fibers (14, 22, 23). In this study, our goal was to analyze the degree to which the more resource-intensive approaches such as DKI, 7T, or computationally expensive processing bring benefits in terms of increased sensitivity to WM pathology in patients with SLE, and analyze the degree to which the results align with other studies on different dMRI protocols and processing pipelines.

Concerning the diffusion protocol, the main difference between DTI and DKI is that the latter allows for the estimation of the mean, axial, and radial kurtosis (MK, AK, and RK, respectively) in addition to the parameters obtained with DTI (MD, AD, RD, and FA) (24). The kurtosis parameters characterize the diffusional heterogeneity that might be present in tissues consisting of compartments with different diffusivities (18, 25, 26). This benefit comes at a cost: DKI needs a multi-shell acquisition protocol with at least two non-zero and different *b*-values, in contrast to DTI where a single-shell acquisition is sufficient (27). Moreover, DKI requires the acquisition of images with higher *b*-values (in the range of 2,000–2,500 *s*/*mm*^2^). This, in turn, necessitates diffusion encoding with longer gradient pulses, and therefore, DKI is performed with longer echo times than DTI, which reduces the baseline signal-to-noise-ratio (SNR). This is often compensated for by reducing the spatial resolution in DKI compared with DTI. DKI and DTI have been applied together before (28–38). Generally, these studies point to a rise in FA, MK, AK, and RK and a decrease in MD, AD, and RD in the early development of cerebral WM (29, 30). Then a reverse process takes place later either due to aging (28) or due to neurodegenerative disease in conditions such as schizophrenia (32), MS (31), Alzheimer's disease (33, 34, 36, 37), and Parkinson's disease (38, 39). However, it is not clear whether DTI or DKI is most sensitive to WM pathology (40). For example, in Alzheimer's disease, MD and MK seem to be most sensitive, but some studies highlight the former [e.g., (41)] and others the latter [e.g., (42)]. Overall, MD is often reported to have high sensitivity to neurodegeneration, followed by MK and to a lesser degree FA, RD, and RK (40).

How the magnetic field strength influences the sensitivity of dMRI to pathology has been less extensively investigated than the effect of the diffusion protocol, possibly because ultra-high-field (UHF) imaging (e.g., 7T) has only recently become relatively widely available for clinical research (43–45). For similar image resolution, 7T-DTI offers increased contrast-to-noise-ratio and SNR compared to 3T-DTI (46–48). However, 7T-DTI suffers from increased spatial heterogeneity in brain regions such as the temporal lobes (49, 50). A recent investigation on the impact of the magnetic field strength in a small population of seven MS patients and six healthy controls (HC) showed that both 3T and 7T are viable options for imaging WM tissue change in MS (31).

Apart from the diffusion protocol and the magnetic field strength, image processing can also affect sensitivity to pathology (20, 51–53). Optimizing the processing pipeline has the potential to increase the sensitivity to pathology (54–57). For example, age-related WM changes seem to be best revealed when a combination of all the state-of-the-art processing steps are applied (20).

In this study, we investigated the dependence of the sensitivity to WM pathology in patients with SLE on the diffusion protocol (DTI or DKI), the magnetic field strength (3T or 7T), and the inclusion of various processing steps (denoising, Gibbs-ringing removal, eddy-current and motion correction, and smoothing, in different combinations). Data was acquired with three protocols: 3T-DTI, 3T-DKI, and 7T-DTI. The hypothesis was that 3T-DKI and 7T-DTI would show benefits compared with 3T-DTI, as these protocols are more resource-intensive in terms of either time (3T-DKI) or the use of a scarce but SNR-boosting resource (7T-DTI). We also hypothesized that a more extensive and, thus, computationally intensive processing pipeline that incorporates several state-of-the-art processing steps would be beneficial. To test these hypotheses, we performed a region-based effect-size analysis. Cohen's *d* was used as a measure of effect size, as it evaluates the difference in means between two populations normalized by their joint SD (58). The effect size analysis was applied to analyze the difference between patients with SLE and HC in 72 major WM fiber tracts obtained from TractSeg (59, 60).

## 2. Materials and Methods

**Figure 1:**
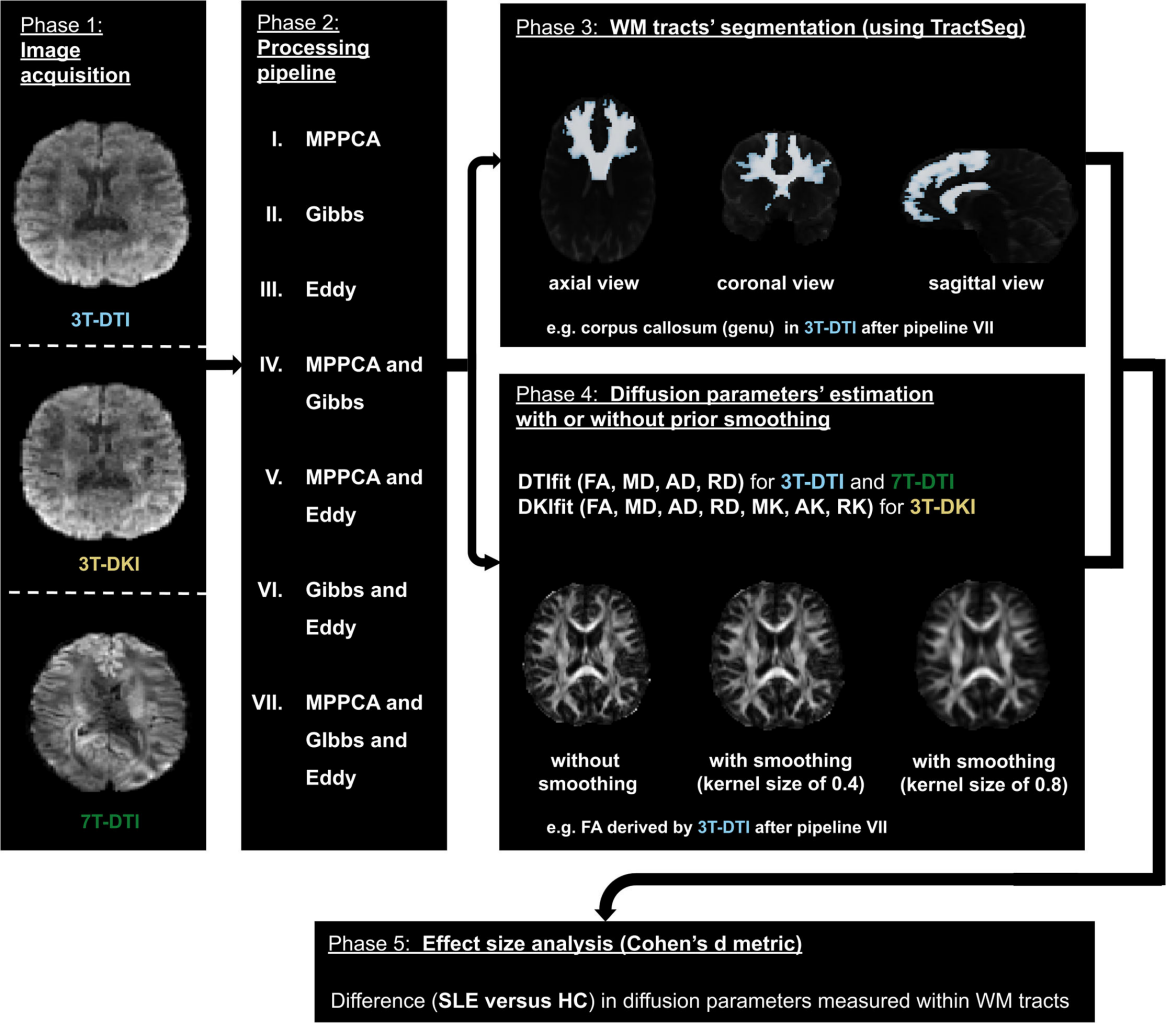
The workflow. The diffusion MRI (dMRI) data were acquired using three different acquisition protocols: 3T-diffusion tensor imaging (DTI), 3T-diffusion kurtosis imaging (DKI), and 7T-DTI (left- most block). The three slices in the left block correspond to the same axial view of the dMRI volume with a *b*-value of 1,000 *s*/*mm*^2^ in each of the three protocols. Subsequently, dMRI data were processed by seven different processing pipelines (a second block from the left). The processed data were then used by TractSeg to segment 72 major white matter (WM) tracts (top right most block). The processed dMRI data were also fitted using either DTI or DKI (depending on the initial acquisition) to extract dMRI parameters. To assess the impact of smoothing prior to model fitting, this fitting step was performed with different degrees of smoothing, ranging from no to substantial smoothing (right most block second from top). Finally, Cohen's *d* was computed from the average and SD of the parameters within the 72 WM tracts segmented earlier (bottom block).

Figure 1 shows the workflow of this study. In this section, we describe each of those steps from data acquisition to effect size estimation.

### 2.1. Data Acquisition and Participants

Imaging was performed on two different systems (3T Siemens Skyra and 7T Philips Achieva) with three different protocols, referred to as 3T-DTI, 3T-DKI, and 7T-DTI. The image resolutions, *b*-value scheme, and repetition and echo times (TR and TE) were adjusted for each protocol and system independently, and are reported in Table 1. Generally, TE and TR were minimized. The resolution and other imaging parameters were adjusted to minimize artifacts such as signal bias due to the rectified noise floor (61). Specifically, as 3T-DKI includes the acquisition of high *b*-value data with lower SNR, the 3T-DKI was performed with a lower resolution than the 3T-DTI protocol in order to avoid noise-floor effects. A 7T-DKI protocol was not included due to limitations in total scan time per patient. The 7T-DTI protocol featured fewer encoding directions than the 3T-DTI protocol, also due to scan time prioritizations. The extent to which these protocol differences affected the results will be considered in the discussion.

**Table 1:** Demographics and image acquisition parameters.

**Image acquisition**	**#SLE, #HC**	**Mean age (std) of HC, SLE**	**Image resolution (isotropic, in *mm*^3^)**	***b*** **-values in *s*/*mm*^2^ (# of directions)**	**TR/TE in ms/ms**
3T-DTI	63, 20	37 (9), 36 (9)	2.0	0 (8), 1,000 (64)	7,300/73
3T-DKI	56, 20	37 (9), 35 (9)	2.3	0 (3), 250 (6), 500 (6), 1,000 (20), 2750 (30)	7,500/103
7T-DTI	54, 21	40 (10), 40 (9)	2.0	0 (3), 1,000 (30)	8,816/62

*Rows show the three protocols: # corresponds to the number of, SLE, patients with systemic lupus erythematosus; HC, human controls; TR, repetition time; TE, echo time. In total 106 subjects participated in this study: 76 patients with SLE and 30 HC. Out of the 106 subjects, 47 were scanned with all three image acquisition protocols (3T-DTI and 3T-DKI and 7T-DTI): 37 patients with SLE and 10 HC*.

In total, 106 female subjects were scanned. Out of these, 76 were SLE patients and 30 HC. The Regional Ethical Review Board in Lund, Sweden approved the studies on 3T and 7T (#2012/254, #2014/778, #2016/30, #2019/01953) and all participants gave written informed consent prior to the examinations. None of the controls had a history of neurologic, neurodegenerative, or psychiatric disorders. The 106 subjects were investigated with at least one of the acquisition protocols (refer to **Table 3**). Out of the 30 HC, 13 were scanned with all the three different protocols, 20 with at least 3T-DTI, 20 with at least 3T-DKI, and 21 with at least 7T-DTI. Out of the 76 patients with SLE, 59 were scanned with all the three acquisition protocols, 63 with at least 3T-DTI, 56 with at least 3T-DKI, and 54 patients with at least 7T-DTI.

### 2.2. Processing Pipeline

To analyze the effect of the processing pipeline on the effect size in a group comparison, we built seven processing pipelines. These comprise some or all of three processing steps: denoising, correction for Gibbs-ringing artifacts, and correction of distortions due to head motion and eddy currents. For denoising, we used the method proposed by Veraart et al. (73), termed Marchenko-Pastur principal component analysis (MPPCA). This method is based on the idea of applying principal component analysis (PCA) within a local neighborhood of the voxel, in order to shrink the redundant components over which thermal noise is spread and instead reveal the signal-carrying principal components (74). In contrast to previous local PCA denoising approaches by Manjón et al. (75), MPPCA automatically estimates the number of eigenvalues associated with noise by using random matrix theory for noisy covariance matrices (76). For removal of Gibbs-ringing artifacts, which appear due to a k-space truncation (77), we used the method presented in Kellner et al. (78). In that study, Gibbs-ringing artifacts, most often appearing on sharp edges, are minimized by finding the optimal subvoxel-shift for pixels in the neighborhood of such sharp edges. Finally, dMRI data also suffer from subject motion as well as eddy current-induced artifacts due to the strong and rapidly switching diffusion encoding gradients. To correct for such distortions, we used eddy from FSL (79).

The seven pipelines that were examined in this study consisted of *MPPCA* for denoising, *Gibbs* for removal of Gibbs-ringing artifacts, *Eddy* for correction of motion and eddy current-induced distortions, *MPPCA and Gibbs* in combination, *MPPCA and Eddy* in combination, *Gibbs and Eddy* in combination and *MPPCA and Gibbs and Eddy* in combination (refer to phase 2 in Figure 1). For reference, we also investigated the effect of applying no processing at all (i.e., *none*). We also evaluated the impact of Gaussian smoothing, by smoothing the diffusion-weighted imaging data using kernels with SDs ranging from 0 to 1 in units of 0.1 (refer to phase 4 in Figure 1). The purpose of smoothing is to increase SNR prior to the estimation of diffusion scalar metrics (20). All three processing methods (MPPCA, Gibbs, and Eddy) were incorporated in recently published articles (20, 52) and smoothing has often been applied in studies involving DTI or DKI (20, 39, 68, 80–84).

### 2.3. Segmentation of WM Tracts

To obtain tract-specific parameter values, WM tract segmentation was performed using TractSeg (59, 60). This is a convolutional neural network-based segmentation approach that automatically segments 72 major WM tracts in the native space of the diffusion-weighted images. The algorithm was pretrained on reference segmentations of tracts for 105 subjects from the Human Connectome Project (85). The main benefit of TractSeg is that it is both fast and accurate (60). It achieves that by directly segmenting the tracts in the field of fiber orientation distribution function peaks without using tractography and image registration. A list of all 72 tracts can be found online (https://github.com/MIC-DKFZ/TractSeg).

### 2.4. Diffusion Parameter Estimation

**Figure 2:**
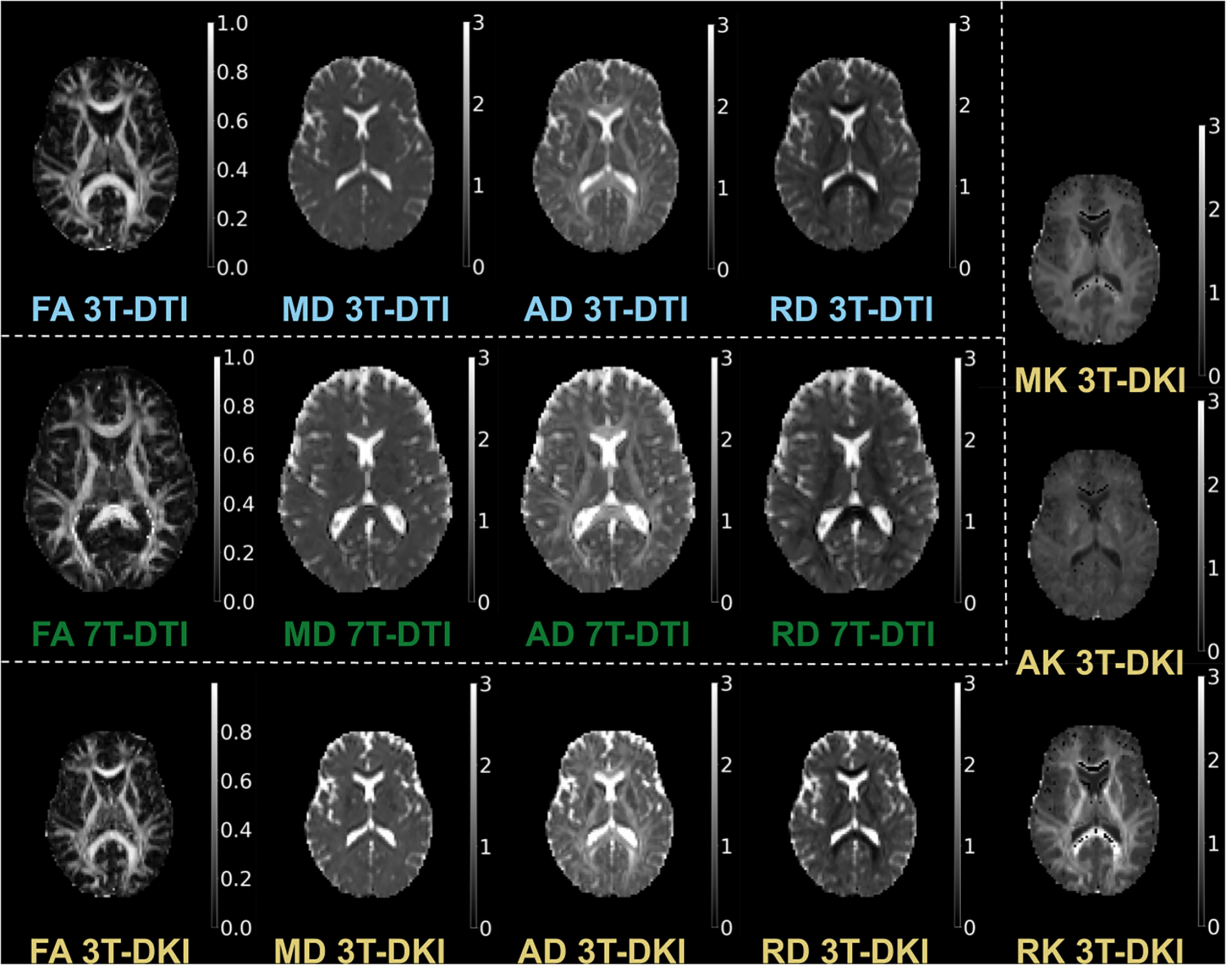
Examples of parameter maps. The maps derived by 3T-DTI are labelled in cyan, the ones derived by 7T-DTI in green, and the ones derived by 3T-DKI in gold color.

We estimated the diffusion parameters using two approaches: DTI (86), which provided FA, MD, AD, and RD, and DKI (18), which provided MK, RK, and AK in addition to the parameters provided by DTI. For DTI, we used DTIFIT in FSL with weighted linear least squares. For DKI, we used the package dipy and its module *DiffusionKurtosisModel*, again with weighted linear least squares. Examples of parameter maps are shown in Figure 2.

### 2.5. Effect Size Estimates

We evaluated for each tract and diffusion parameter the groupwise difference between SLE patients and HC by Cohen's *d* (58), which provides an effect size measure (87) defined as:


(1) $$\begin{array}{r} d=\left(u_{HC}-u_{SLE}\right)/s \end{array}$$

with


(2) $$\begin{array}{r} s=\sqrt{\left(\left(n_{HC}-1\right)s_{HC}^{2}+\left(n_{SLE}-1\right)s_{SLE}^{2}\right)/\left(n_{HC}+n_{SLE}-2\right)} \end{array}$$

where u_*HC*_ and u_*SLE*_ are the mean values of the parameters within the tract for the HC and SLE groups, respectively, and s is the pooled SD of the two groups. Moreover, *n*_*HC*_ and *n*_*SLE*_ are the sizes and $s_{HC}^{2}$ and $s_{SLE}^{2}$ the variances of the HC and SLE groups, respectively. To make it easier to compare effect sizes across parameters, we changed the sign of the effect size estimate in MD, RD, and AD so that all parameters had positive effect size estimates. As Cohen's *d* is a standardized effect size measure, it can be more easily compared across studies and populations (88). Of note, for an unbalanced dataset like ours (more patients than controls), the pooled SD in the above Cohen's *d* formula mostly reflects the SD of the larger group (i.e., the patients), which tends to be more heterogeneous and, thus, have higher SD than the controls group. This will, in turn, provide lower effect sizes than was would have been observed in a balanced setting. However, this does not affect the comparison of effect sizes, which is the main topic of the study.

To assess statistical significance, we note that the 95% CI for Cohen's *d* in the absence of a true effect (a true d of zero) for group sizes of 56 and 20, as in 3T-DKI spans the range where the magnitude of d is below 0.54. Effect sizes larger than this can, thus, be considered significant on a 5% significance level (89). In the case of both 3T-DTI and 7T-DTI, the level of significance was set to 0.53, based on the corresponding sizes of the cohorts. Effect sizes were estimated in 72 tracts and, thus, multiple comparison problem arises. We did not correct this, but note that a Bonferroni-like correction can be applied (90–92). Correcting for 72 independent tests corresponds to using the 99.9% CI (1 - 0.05/72), which in turn corresponds to a minimum threshold for a significant effect at *d* = 0.84. Another way to approach the multiple comparisons problem is to note that in the absence of a true effect in all 72 tracts, the probability to still identify more than seven tracts as significant on the uncorrected significance level (5%) is less than 5% (93). This means that observing more than seven tracts as significant on the uncorrected level indicates a true effect in at least some of those tracts.

## 3. Results

The results that are primarily reported have been derived from comparisons using all subjects, but complementary analyses were also performed using only subjects with data from all three protocols. These latter results are reported in the Supplementary Material. For the effect size analysis using Cohen's *d*, we computed mean and SDs of each diffusion parameter (e.g., FA), that were weighted by each tract probability-mask. However, before the above multiplication, we first excluded the voxels that in the probability map had a probability value of less than 0.5.

### 3.1. WM Fiber-Tracts Segmentation

**Figure 3:**
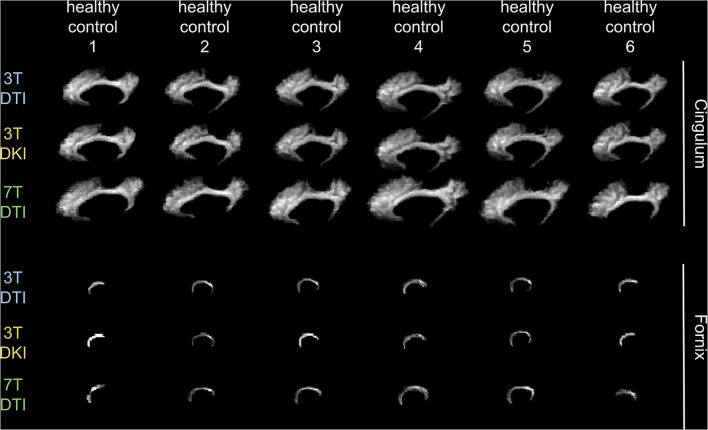
Demonstration of how a tract's volume change across six exemplar human controls for each of the three examined acquisition protocols. We chose to present the variation in the cingulum (top 3 rows) and in the fornix (bottom three rows), as an example of a tract that does not and does, respectively, challenge TractSeg in segmenting it. Note that the fornix is a very small tract in contrast to the cingulum that has a more recognizable shape, faciliating in that way TractSeg on segmenting it and vice-versa for the fornix.

**Figure 4:**
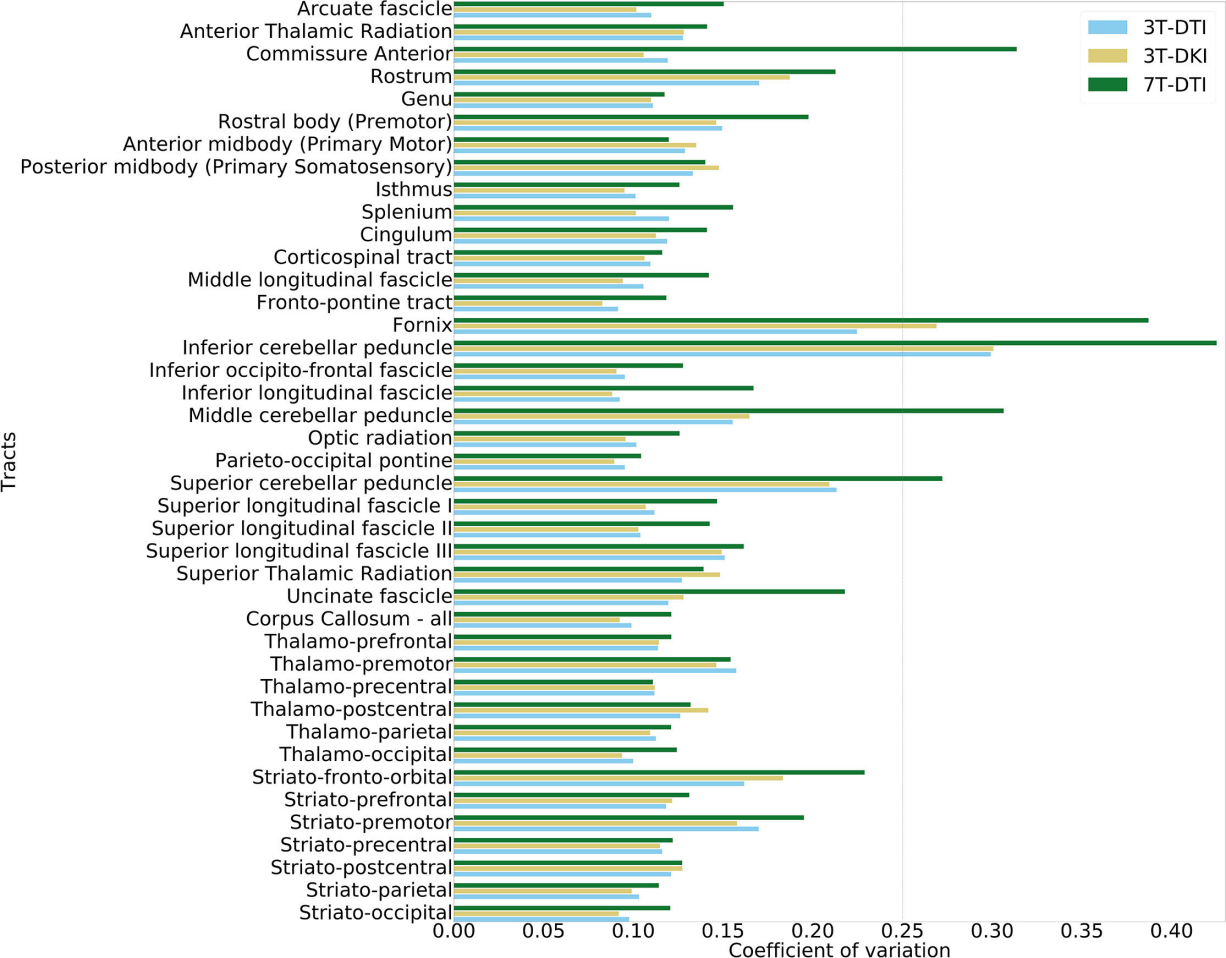
Evaluation of volume variation in the tracts. The tract segmentation was based on data acquired with 3T-DTI (cyan), 3T-DKI (gold), and 7T-DTI (green) data. In cases where TractSeg provides the left and right part of one fiber-tract as two different tracts (e.g., in case of left and right arcuate fasciculus), the left and right parts of the tract were averaged. The vertical dashed line shows the threshold of 0.25, which corresponds to a high variation.

Figure 3 shows example segmentations of the cingulum and the fornix across different subjects. While the cingulum was segmented consistently, a large variation in segmentation performance is seen for the fornix. Figure 4 shows the segmentation performance for all tracts in terms of the coefficient of variation of the tract volume. The analysis was applied to HC only, as we expect the least variation in that cohort (Figure 4). The values were averaged for tracts found in both the left and right hemispheres, resulting in 41 rows. Three tracts exhibited excessively high variation in tract volume for data acquired with all three protocols (the fornix, the inferior cerebellar peduncle, and the superior cerebellar peduncle). For 7T-DTI, another two tracts showed high volume variation (the anterior commissure and the middle cerebellar peduncle). High variation in volume was defined as a coefficient of variation exceeding 0.25, which is considerably larger than expected from pure variation in anatomy (94). For reference, the coefficient of variation of the total brain volume, the total intracranial volume, the total WM volume, and the total gray matter volume extracted *via* MRI volumetry is approximately 0.07, 0.12, 0.08, and 0.07, respectively (95). The excessively large volume variation in the aforementioned tracts indicates that TractSeg struggled to reliably segment these across the cohort. The fornix and the anterior commissures are two of the smallest tracts and are known to be challenging to segment with TractSeg (60), as these are both small structures. The cerebellar peduncles, although larger than the fornix and the anterior commissure, also presented a segmentation challenge probably because the diffusion tensors change dramatically in the region where the peduncles cross (96). Due to the unreliable segmentation, these five tracts were excluded from further analysis (i.e., the fornix, commissure anterior, superior, inferior, and middle cerebellar peduncles). Note that high volume variation was associated mainly with 7T, as 7T-DTI gave a higher volume variation among the three protocols in most of the tracts regardless of the choice of the pipeline (results shown in Figure 4 were derived by pipeline VII, however, highly similar results were obtained with all seven pipelines). Some possible explanations for this are given later in the discussion.

### 3.2. Effects of Individual Pipelines

**Figure 5:**
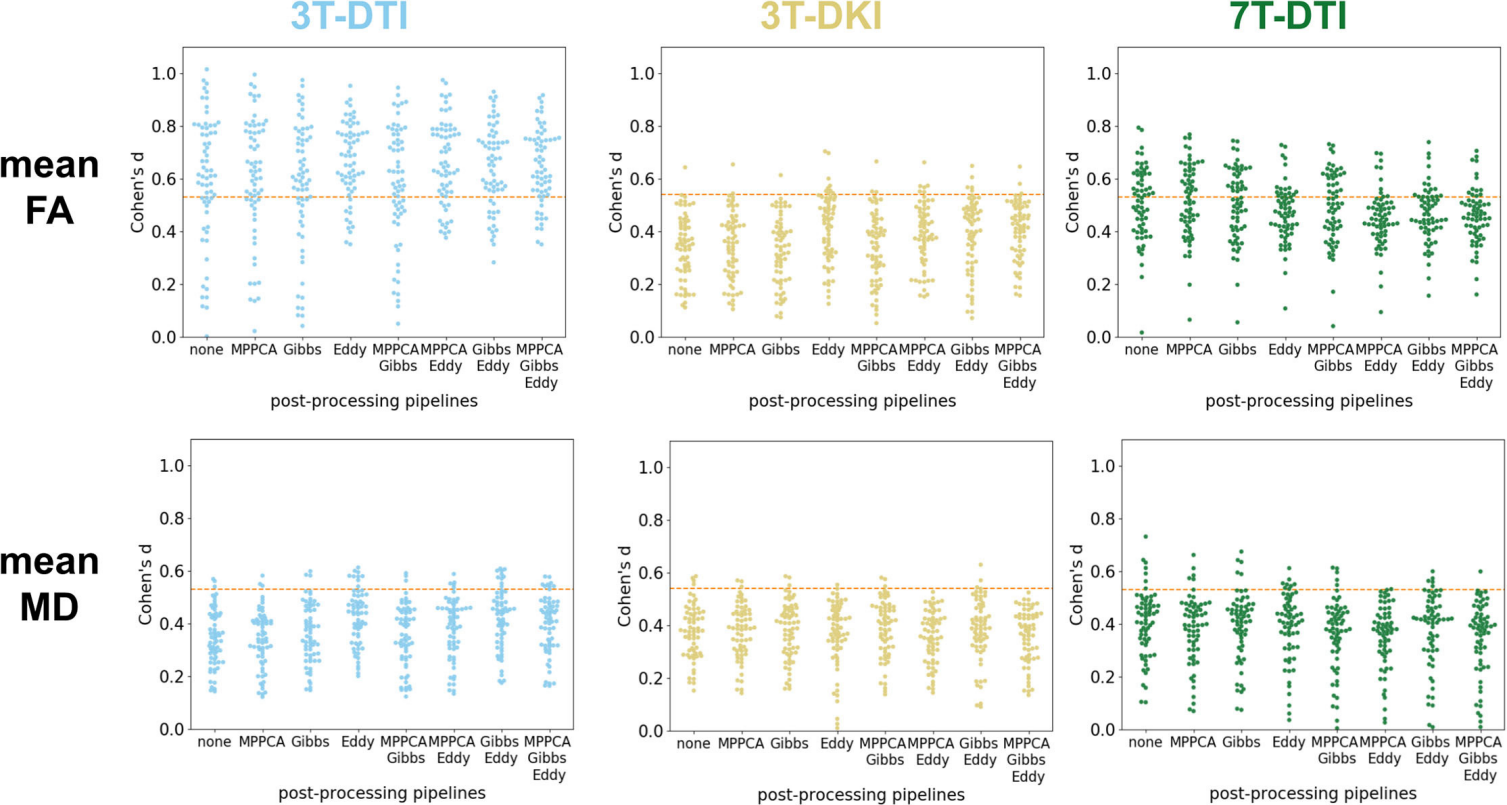
Impact of the processing pipeline. The impact of the processing pipelines (x-axis) is evaluated through effect size estimates (Cohen's *d* scores, y-axis). Top row: effect size estimates in mean fractional anisotropy (FA) from 3T-DTI (top left), 3T-DKI (top center), and 7T-DTI (top right). Bottom row: effect size estimates with mean diffusivity (MD) from 3T-DTI (top left), 3T-DKI (top center), and 7T-DTI (top right). No smoothing was applied. Each dot represents one tract. An orange dotted line in each plot defines the threshold in effect size above which the result is considered significant.

Figure 5 shows effect size estimates for different processing pipelines. Rows show results for different parameters (mean FA and mean MD), while columns show results from the three protocols (3T-DTI, 3T-DKI, and 7T-DTI). The figure shows that the choice of processing pipeline had a smaller effect than the choice of acquisition protocol. MPPCA on average (across all tracts and acquisition protocols) reduced the effect sizes (by 0.005, 0.018, 0.014, and 0.018 for FA, MD, AD, and RD, respectively). However, MPPCA had a positive impact on all diffusion kurtosis parameters, with the strongest effect in RK (+0.023). Gibbs-ringing removal had a mixed impact on the effect sizes for DTI parameters (-0.010, +0.003, +0.023, and -0.007 for FA, MD, AD, and RD, respectively), but a positive effect on all DKI parameters related to diffusivity (MD, AD, and RD), with the highest increase seen in AD (+0.022). Finally, eddy current and motion correction on average had a positive effect on all DTI parameters and increased their effect sizes by 0.031, 0.0003, 0.006, and 0.010 for FA, MD, AD, and RD, respectively. Table 2 shows another overview of the influence of the processing steps on effect sizes, where the effect of each specific step can be judged by comparing pipelines with and without that step. Overall, the incorporation of eddy current and motion correction increased the effect size by 0.016 on average, while the incorporation of Gibbs-ringing removal and MPPCA reduced effect sizes by 0.005 and 0.011 on average, respectively.

**Table 2:** The impact of each processing step (Marchenko-Pastur principal component analysis (MPPCA), Gibbs, and Eddy) on the effect size.

**Parameter**	**Protocol**	**MPPCA**	**Gibbs**	**Eddy**
FA	3T-DTI	–0.005	–0.028	+0.069
	3T-DKI	–0.003	–0.013	+0.059
	7T-DTI	–0.009	–0.017	–0.039
MD	3T-DTI	–0.026	+0.009	+0.068
	3T-DKI	–0.011	+0.006	0.000
	7T-DTI	–0.029	–0.015	–0.013
AD	3T-DTI	–0.019	+0.027	+0.029
	3T-DKI	–0.011	+0.018	–0.021
	7T-DTI	–0.020	+0.004	+0.022
RD	3T-DTI	–0.026	–0.010	+0.081
	3T-DKI	–0.010	–0.005	+0.021
	7T-DTI	–0.024	–0.020	–0.026
MK	3T-DTK	+0.020	–0.025	–0.022
AK	3T-DKI	+0.005	–0.002	+0.016
RK	3T-DKI	+0.010	–0.002	–0.009
Mean effect over all parameters	Over all protocols	–0.011	–0.005	+0.016

*A positive value denotes an increase in effect size, whereas a negative value denotes a decrease. The rows correspond to the mean of each diffusion parameter*.

**Figure 6:**
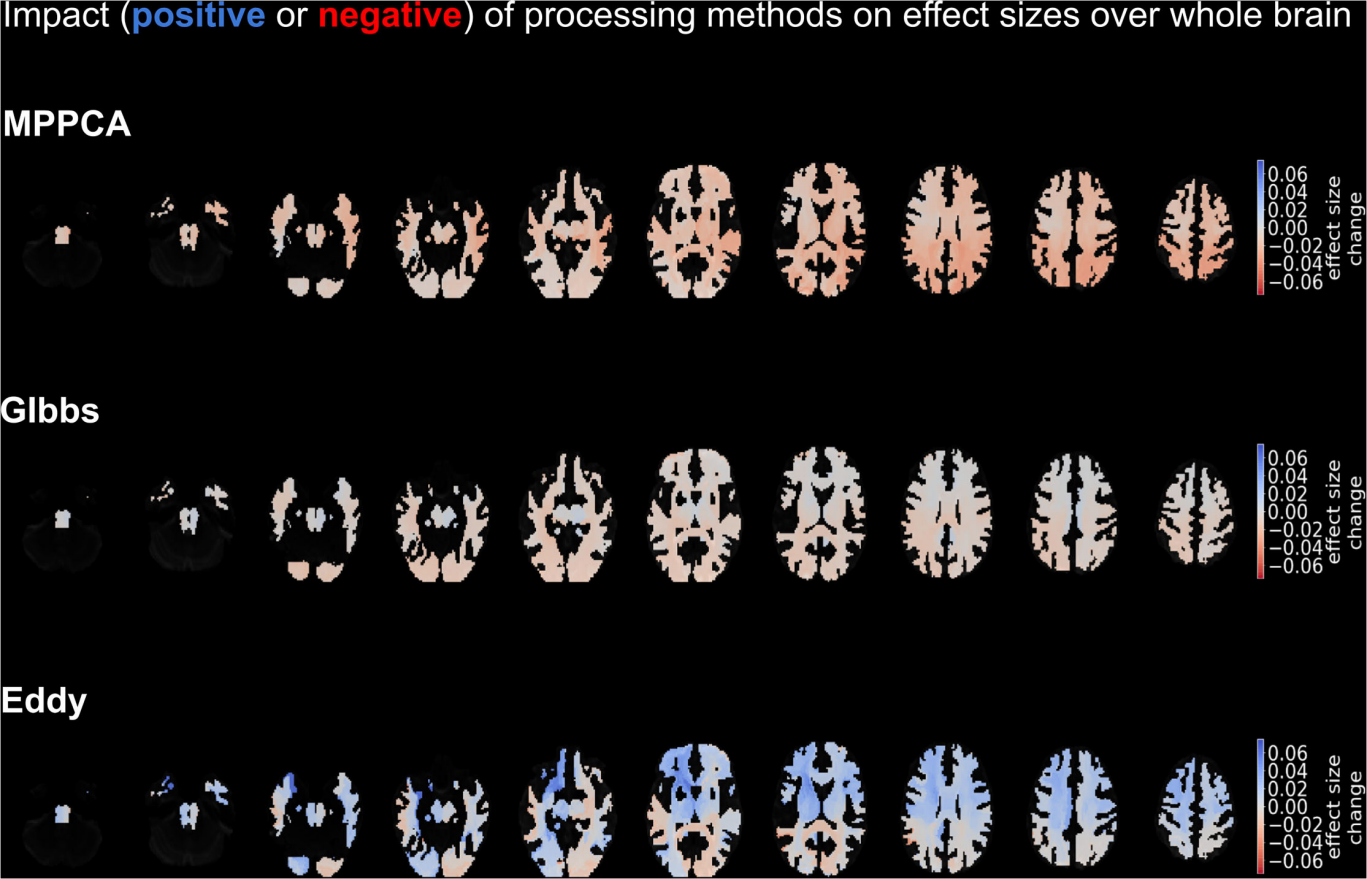
Depiction of the impact on effect sizes of each processing method. Impact is demonstrated in shades of blue (positive impact) and red (negative impact), with the former colors denoting an increase in effect size on that tract due to the method and the latter a decrease in effect size due to the method. The top panel refers to the impact of MPPCA method, the middle panel to the impact of Gibbs method, and the bottom panel to the impact of Eddy method. The depicted results were obtained by averaging over the mean diffusion tensor and kurtosis parameters, as well as the acquisition protocols.

Figure 6 depicts the change in effect sizes over the whole brain due to each method. Among all tracts, the strongest negative impact of MPPCA on the effect size was seen in the isthmus, the middle longitudinal fascicle, and the thalamo-parietal (top panel of brains in Figure 6). In these tracts, effect sizes decreased by more than 0.04 due to MPPCA. Moreover, the inclusion of MPPCA did not lead to a substantial increase in effect sizes in any of the tracts. The inclusion of Gibbs removal caused a slight increase in effect size in the corticospinal tracts (+0.018) and had a less detrimental effect on effect sizes in general compared with MPPCA. The inclusion of Eddy reduced effect sizes in the splenium and the left middle longitudinal fascicle by more than 0.03. However, it also increased effect sizes by more than 0.06 in the left striato-fronto-orbital, the left thalamo-posrcentral, and the left striato-premotor (bottom panel of brains in Figure 6).

Overall, Eddy was the most impactful processing step. Therefore, we performed the subsequent analysis with a pipeline that included Eddy. Four pipelines met that criterion (pipelines III, V, VI, and VII). In the end, we chose pipeline VII, which combines all three tested processing steps, since that pipeline was the closest among the four to the most preferable processing scheme in the literature (20, 52).

### 3.3. Effect of Smoothing

**Figure 7:**
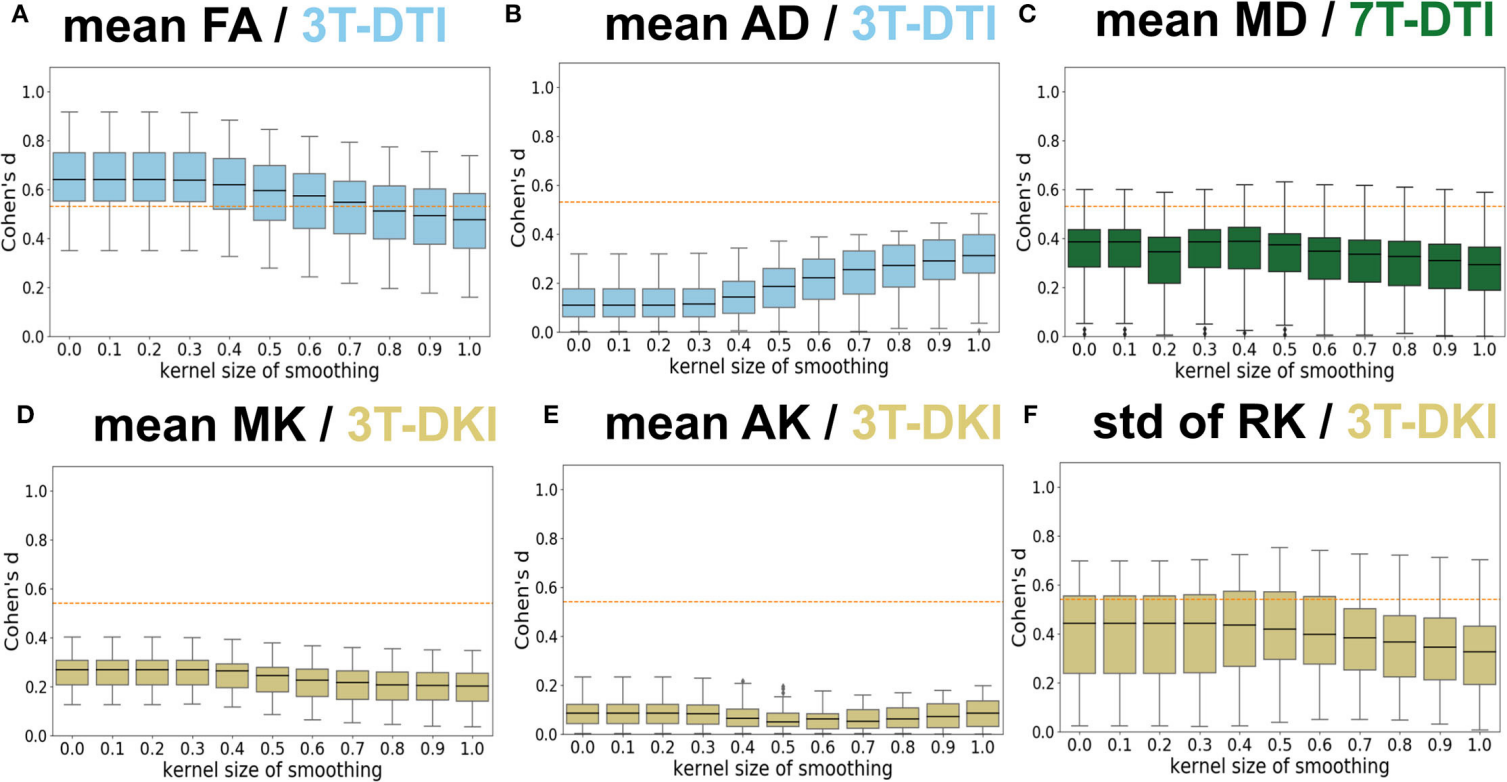
Influence of Gaussian smoothing. Different levels of Gaussian smoothing (sigma, x-axis) were evaluated by the effect size estimates (Cohen's *d*, y-axis). **(A–F)** shows the effect on mean FA from 3T-DTI, mean axial diffusivity (AD) from 3T-DTI, mean MD from 7T-DTI, mean MK from 3T-DKI, mean AK from 3T-DKI, and SD of RK from 3T-DKI. The box plots show Cohen's *d* scores among tracts. Overall, smoothing had a detrimental influence on effect sizes. An orange dotted line in each plot defines the threshold in effect size above which the result is considered significant.

Figure 7 shows the influence of smoothing on the effect size. Four main patterns were identified. The first and dominant pattern comprised a decline in effect size with greater smoothing. This pattern applied to the mean FA (Figure 7A), the SD of FA, the mean MD (Figure 7C), the SD of AD, and the mean MK (refer to Figure 7D) and RK. The second pattern was one where smoothing had little influence on the effect size and applied to the SD of MD, the mean and SD of RD, and the mean AK (refer to Figure 7E). The third pattern was one where the effect size increased slightly with greater smoothing up to a kernel with a SD of 0.4–0.5 followed by a decline for greater smoothing. This applied to the SD of the three DKI parameters (MK, AK, and RK, refer to Figure 7F). The fourth pattern comprised a slight increase with smoothing and was noted only for the case of mean AD (refer to Figure 7B). Note that the smoothing only affected parameter estimation but not the tract segmentation as smoothing was applied after TractSeg and before parameter estimation. This analysis suggests that smoothing was overall detrimental for the effect sizes for all three protocols and, therefore, subsequent analyses were performed without smoothing.

### 3.4. Effects of Magnetic Field Strength and Acquisition Protocol

**Figure 8:**
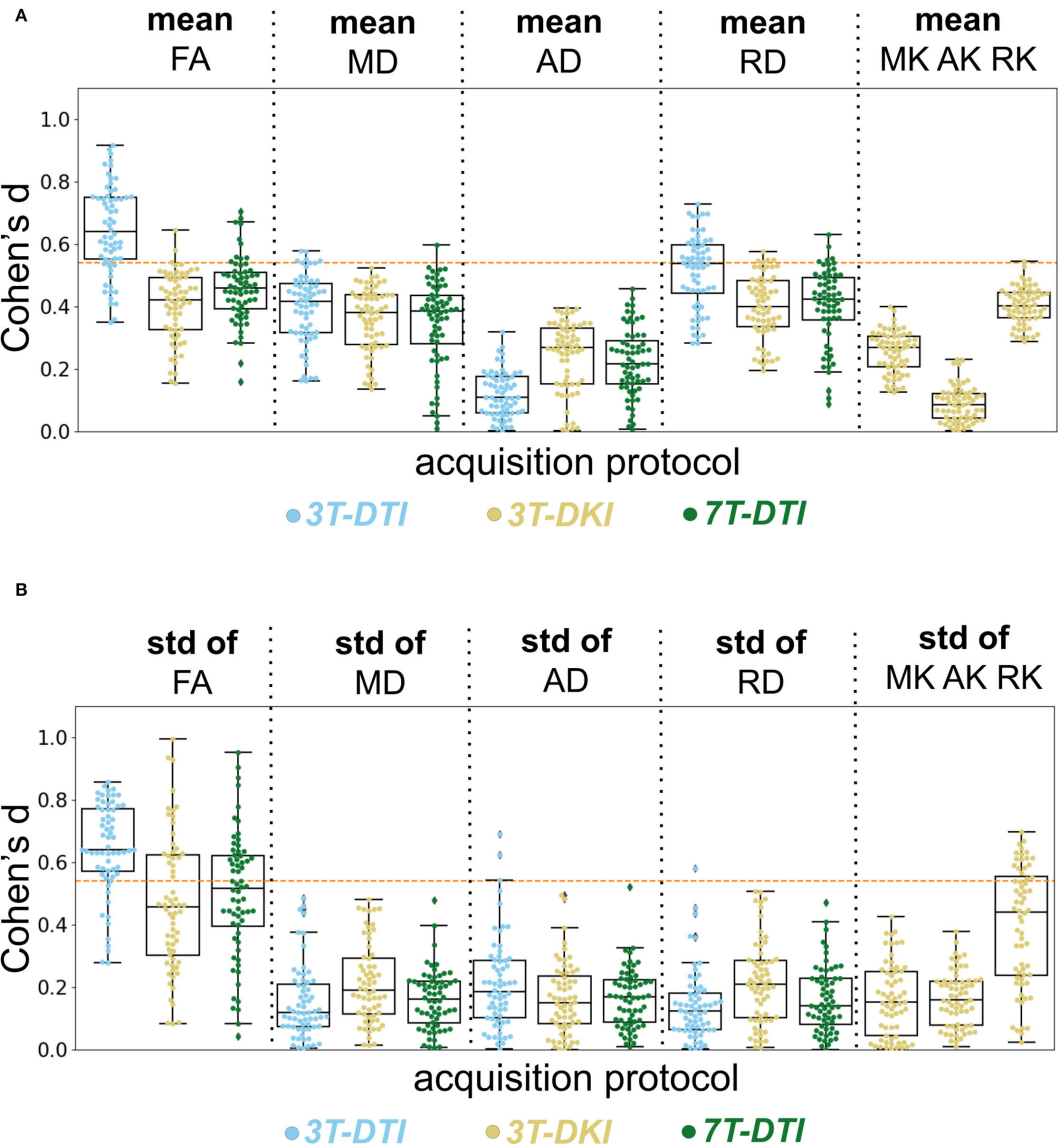
Evaluation of the acquisition protocol on the effect size. Columns show different diffusion parameters obtained with different protocols 3T-DTI, 3T-DKI, or 7T-DTI, shown in cyan, gold, and green, respectively. The mean **(A)** and SD **(B)** of each diffusion parameter, derived for each protocol were examined. Pipeline VII was used in all cases. No smoothing was applied. The swarm and box plots show the distribution and quartiles (together with the median) of Cohen's *d* values among tracts, respectively. Overall, 3T-DTI was the acquisition protocol that yielded the three most sensitive diffusion parameters [mean FA, SD of FA, and mean radial diffusivity (RD)], and exhibited effect sizes above 0.54 a threshold of significance before correcting for multiple comparisons) in the largest number of tracts. Moreover, the results between 3T-DTI and 7T-DTI were more similar than those between 3T-DTI and 3T-DKI, which implies that the choice of the diffusion protocol impacted the effect size analysis more than the choice of the magnetic field strength. Kurtosis parameters did not yield significant effect sizes, with the exception of the SD of RK, which had significant effects in a small number of tracts. An orange dotted line in each plot defines the threshold in effect size above which the result is considered significant.

Figure 8 shows effect sizes for data acquired with different diffusion protocols (DTI and DKI) and different magnetic field strengths (3T and 7T) using the mean (Figure 8A) and the SD (Figure 8B) of the parameters within the tracts.

Among all parameters from all protocols, the three that had the largest number of tracts with an effect size above 0.54 (significant before correction for multiple comparisons) were the mean and SD of FA and the mean RD, all derived by 3T-DTI. 3T-DTI yielded clearly higher effect sizes than 3T-DKI and 7T-DTI for the mean FA, with an average difference of 0.26 and 0.20, respectively. The FA was the parameter with the highest effect size overall. Among the DKI parameters, RK showed on average the largest effect sizes (refer to far right plots of Figures 8A,B).

**Figure 9:**
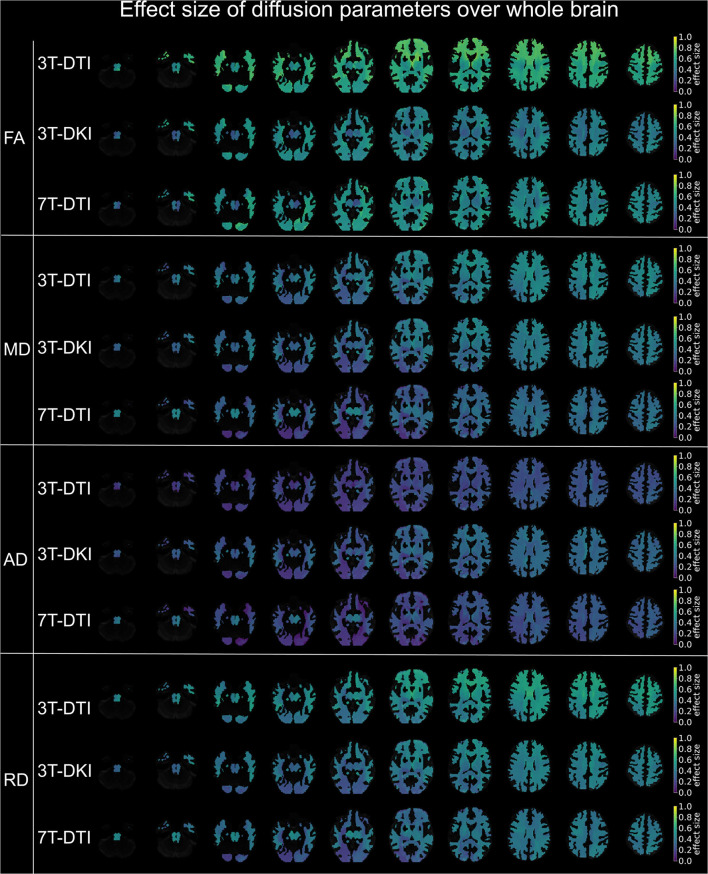
Effect size of diffusion tensor parameters (FA, MD, AD, and RD) over the whole brain, when derived by the different acquisition protocols (3T-DTI, 3T-DKI, and 7T-DTI). Effect sizes in the depicted brains range from 0.0 (deep purple areas) to 1.0 (yellow areas). Overall, FA and RD were the parameters with the highest effect sizes across all three acquisition protocols, but the FA derived by 3T-DTI was the diffusion indices with the highest number of areas in the brain exceeding the value 0.54 of effect size. In the case of FA, 3T-DTI (top panel, first row) yielded the highest number of tracts with significantly large effect size than 3T-DKI (top panel, second row) and 7T-DTI (top panel, third row) since the brains in the top panel appear brighter than the brains in the second and third panels.

**Figure 10:**
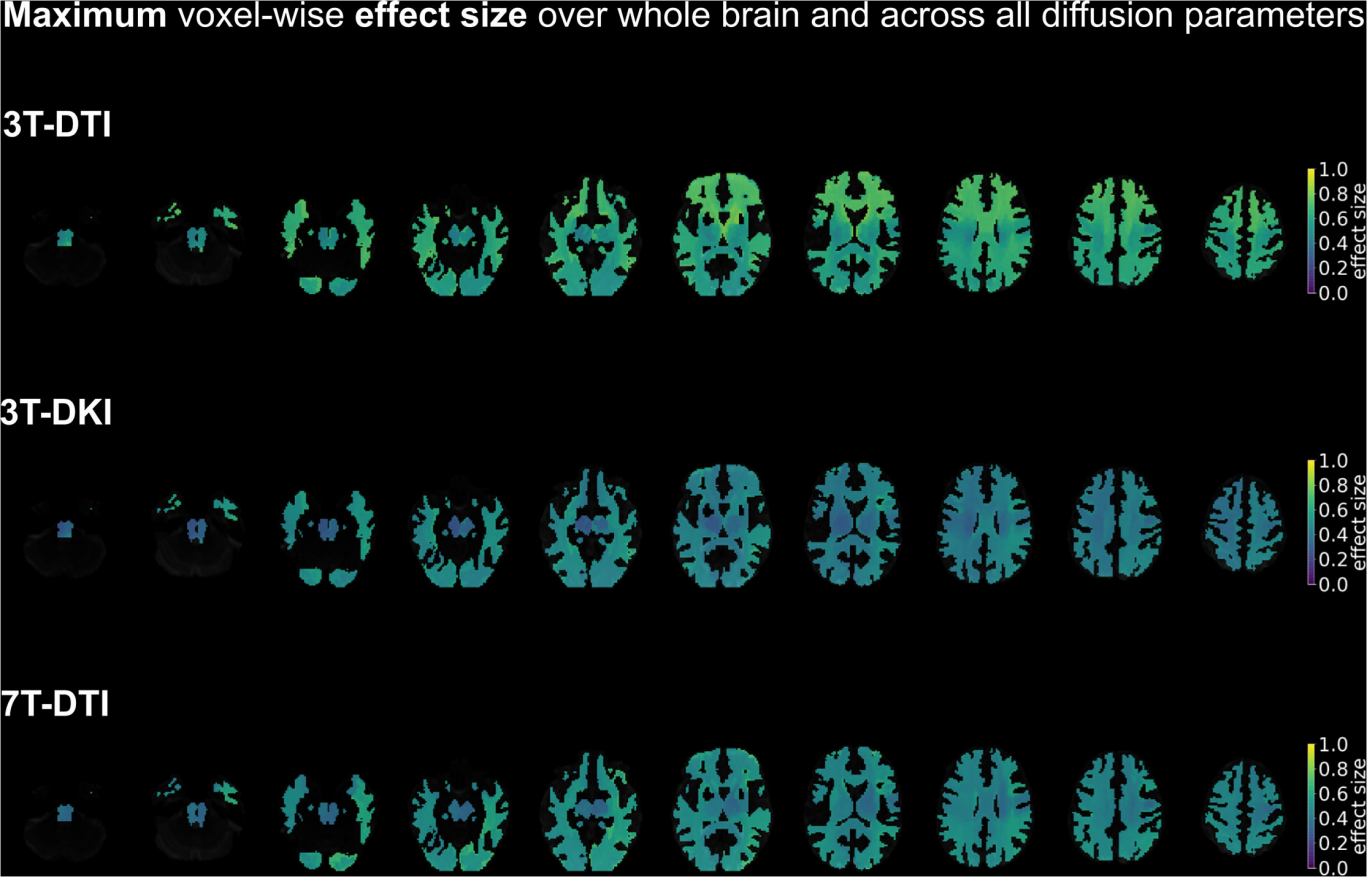
A summary of the spatial distribution of effect sizes over the whole brain, taking the maximum voxel-wise effect sizes across all diffusion tensor and kurtosis parameters (FA, MD, AD, and RD for all three acquisition protocols plus MK, AK, and RK in case of 3T-DKI). Effect sizes in the depicted brains range from 0.0 (deep purple areas) to 1.0 (yellow areas). Overall, 3T-DTI was the protocol with the highest effect sizes among all three acquisition protocols, with more voxels with an effect size exceeding the value 0.54, which was the threshold for significance (turquoise colors).

Furthermore, the within-tract SD of the FA and RK exhibited similar if not higher effect sizes than the mean (Figure 8B). In some tracts, the SD of FA was the only parameter that exhibited effect sizes above the significance threshold. The SD of FA, when derived by the 3T-DTI protocol, was the parameter for which the most tracts showed significant effects (31 out 41 or 76% of all tracts), followed by the same parameter when derived by 7T-DTI and 3T-DKI (51 and 32% of all tracts, respectively; see Figure 8B). In 35 out of the 41 tracts, the effect size from 3T-DTI was higher than that derived by 3T-DKI. Effect sizes from 3T-DTI were also higher than those from 7T-DTI in 37 out of the 41 WM tracts. The fact that 3T-DTI yielded the highest number of tracts with a significant effect size can be appreciated also from Figures 9, 10, where brighter colors indicate higher effect sizes.

Finally, we also evaluated all results using only the subjects that were scanned with all three protocols (refer to Figures 11–15 in Appendix). The outcome of that evaluation was similar to the one presented in this section (3T-DTI yielded on average the highest effect sizes), although the margin between 7T-DTI and 3T-DTI was smaller.

## 4. Discussion

Our analysis of WM pathology in SLE assessed by dMRI showed that the diffusion protocol had the strongest influence on effect sizes among the ones examined: diffusion protocol, magnetic field strength, and processing pipeline. The two DTI protocols (3T-DTI and 7T-DTI) yielded higher effect sizes than the 3T-DKI protocol for most parameters. In only three out of the eight parameter-wise comparisons, did DKI yield higher effect sizes: the mean AD and the SD of MD and RD (refer to Figure 8). Overall, FA was the parameter displaying the highest effect sizes in all the protocols. Using 3T-DTI to compute FA, 76% of all tracts showed significant effects. The corresponding number for 7T-DTI was 51% and 32% for 3T-DKI. The 3T-DKI protocol provided three unique parameters (MK, AK, and RK), but these generally showed low effect sizes. Only RK exhibited effect sizes as high as any of the DTI parameters. These results were consistent across the seven tested processing pipelines. Interestingly and opposite to our initial hypothesis, no substantial increase in sensitivity came with the use of more demanding acquisitions (DKI and 7T). Does this finding generalize across pathologies and across variations in the acquisition protocols? This will be discussed below.

Our observation that DKI is less sensitive than DTI to WM pathology in SLE is not in accordance with findings in other pathologies. For example, DKI parameters revealed differences to a broader extent than DTI parameters across age groups (30), between patients with Parkinson's disease and HC (68), and between patients with MS and HC (31). DKI also demonstrated a sensitivity superior to that of DTI in Alzheimer's disease (34) and temporal lobe epilepsy in children (62). On the other hand, MD from DTI showed a greater extent of differences across a WM skeleton than any DKI parameter in subjects with sport-related concussion (35). Overall, there seems to be a discrepancy in findings among different diseases (refer to Table 3). This discrepancy could be due to the specific effect of the pathology on the WM microstructure. For example, any tissue alteration that results in constant FA and MD but different MK would be detected by DKI but not DTI. This could for example happen due to axonal degeneration in regions with high orientation dispersion when the intra and extra axonal water have similar isotropic diffusivities. In such situations, FA would be low due to the orientation dispersion and MD would be insensitive to the axonal water fraction. However, MK would be sensitive as the diffusion heterogeneity would change. Although not considering this situation exactly, Szczepankiewicz et al. (98) considers similar scenarios. Finding a scenario in which FA and/or MD are sensitive while MK is not, as was the case in the present study, is more challenging and may point to another reason as an explanation of our findings.

A second reason for a discrepancy in findings with DTI and DKI could be specific differences in the acquisition protocol. In our case, the image resolution was lower in the DKI protocol than in the DTI protocol (2.3 mm × 2.3 mm × 2.3 mm vs. 2 mm × 2 mm × 2 mm). Voxel size is known to affect group level results for DTI parameters (99). An increase in voxel size results in a decrease in FA and an increase in MD, AD, and RD due to both an increase in partial volume effects and an elevated SNR (100). In this study, we found that synthetically reducing the resolution by smoothing generally reduced effect sizes. Whether DTI is more sensitive than DKI when acquired in the same resolution needs to be further investigated, however, this is not trivial as the high *b*-values employed in DKI attenuate the signal considerably. This often necessitates a reduction in the spatial resolution to avoid the noise floor (101). In turn, this may result in reduced effect sizes. However, as DKI demands a higher baseline SNR than DTI, this is an inherent limitation of DKI. High-performance gradient coils can partially alleviate this limitation by enabling shorter echo times and, thus, higher baseline SNR (102).

**Table 3:** Overview of studies comparing 3T-DTI to 3T-DKI.

**References**	**Pathology**	**DKI resolution in mm**	**Processing pipeline**	**Analysis method**	**Sensitivity metric**	**Most sensitive**	**Comment**
Gao et al. (62)	Epilepsy	2.5 x 2.5 x 2.5	Eddy	TBSS	PASW Statistics (patients vs controls)	DKI	Small number of subjects
Kamagata et al. (38)	PD	3.0 x 3.0 x 3.0	-	TBSS	Student's *t*-test (PD vs. HC)	DKI	DKI useful for evaluating crossing fibers
Zhu et al. (32)	Schizophrenia	2.0 x 2.0 x 2.0	Eddy	TBSS	% of significantly different skeleton-voxels (schizophrenia vs. HC)	none	DKI complementary to DTI
Coutu et al. (28)	aging	2.0 x 2.0 x 2.0	Eddy	TBSS	correlation of diffusion parameters with aging	none	DKI complementary to DTI
Zhang et al. (63)	PD	1.9 x 1.9 x 3.0	-	ROI-based	student's *t*-test (PD vs. HC)	DKI	ROIs manually drawn
Billiet et al. (64)	aging	2.5 x 2.5 x 2.5	Eddy	ROI-based	quadratic correlation coefficients of metrics with age	DTI	ROIs based on population-based template
Lancaster et al. (35)	mTBI	3.0 x 3.0 x 3.0	Eddy	TBSS	significant different skeleton-voxel values (mTBI vs. HC)	DTI	dMRI acquired 6 months after injury
Chen et al. (34)	AD	1.8 x 1.8 x 1.8	Eddy	ROI-based	classification accuracy (AD, HC)	DKI	ROIs manually drawn
Grinberg et al. (30)	aging	1.9 x 1.9 x 1.9	BckgNoise-Eddy	TBSS	Cohen's *d*	DKI	DTI, DKI varied depending on anatomy
Chung et al. (65)	IWM	2.5 x 2.5 x 2.5	MPPCA-Gibbs-Eddy-Outliers	TBSS	voxel-wise correlation with LNS	DKI	MK and AWF the only sensitive parameters
Karlsen et al. (66)	mTBI	2.5 x 2.5 x 2.5	Eddy	TBSS	Welch's *t*-test (mTBI vs. HC)	none	Combined utility of DTI and DKI suggested
Tan et al. (67)	Astrocytomas	2.5 x 2.5 x 6.0	Eddy	ROI-based	*t*-test (patients vs. HC, *via* SPSS)	DKI	Manual estimation of each parameter's value
De Santis et al. (31)	MS	1.5 x 1.5 x 1.5	Eddy	TBSS	ANOVA	DKI	-
Kamiya et al. (68)	PD	3.0 x 3.0 x 3.0	MPPCA-Gibbs-Eddy-B1	TBSS and ROI-based	significant skeleton-points, correlation with age	DKI	Multidimensional diffusion encoding used
Yang et al. (69)	BD	2.0 x 2.0 x 2.0	Eddy	TBSS	Independent-samples *t*-test (BD vs. HC)	DKI	Higher fidelity in widespread regions in DKI than DTI

*TBSS, Tract-based Spatial Statistics; PASW, Predictive Analytics Software; PD, Parkinson's Disease; HC, human controls; Eddy, eddy-current and motion correction; LNS, Letter Number Sequencing; AD, Alzheimer's Disease; mTBI, mild Traumatic Brain Injury; BckgNoise; correct for background noise (70); IWM, Impaired Working Memory; MPPCA, Marchenko-Pastur Principal Component Analysis; Gibbs, Gibbs' artefact removal; Outliers, a processing step to remove outliers in DTI or DKI fitting (71); AWF, Axonal Water Fraction; MS, Multiple Sclerosis; ANOVA, ANOVA statistical package; BD, Bipolar Disorder; B1, corrected for B1 inhomogeneity (72)*.

The field-strength analysis showed that 3T-DTI yielded smaller variation in tract volume and higher effect sizes than 7T-DTI. It could be argued that the latter was due to the shorter scan time of the 7T-DTI protocol, which featured fewer diffusion encoding directions than the 3T-DTI protocol (30 vs. 64, respectively). It, thus, had a slight disadvantage in terms of protocol performance. An additional analysis of a version of the 3T-DTI data subsampled to have only 30 directions did not substantially degrade its performance, however (refer to Figure 17 in Appendix). Note that studies have investigated the effect the number of gradient directions has on the accuracy of direction-sensitive diffusion parameters such as FA, AD, and RD (99, 103, 104). These studies show that above a certain number of directions [approximately 25 (103)] the accuracy in those parameters does not seem to improve substantially from an increased number of encoding directions (99). We, thus, expect both protocols to be equally accurate in terms of parameter estimation. Another aspect that could have contributed to lower effect sizes at 7T-DTI compared to 3T-DTI is the shorter T2* relaxation times at 7T. This reduces the intensity of k-space lines far from the center and, thus, leads to some image blurring already in the image acquisition step, which reduces effect sizes.

Regarding the high values of coefficient variation in tract volume in 7T-DTI, one possible reason could be the higher B1 heterogeneity at higher fields (49). This effect causes low signal intensities especially inferior in the brain and the lateral sides of the insula. This might explain why we notice the biggest difference in variation in volume between 3T and 7T acquisitions in the inferior cerebellar peduncle, the inferior longitudinal fascicle, the uncinate fascicle, and the striato-fronto-orbital tracts (Figure 4). Factors other than B1 homogeneity could also be considered, such as the field-of-view or the number of encoding directions. For example, Güllmar et al. (105) reported that the size ratio of the structure to-be-segmented and the size of the input samples (field-of-view) might have an effect on the performance of TractSeg. An additional analysis on the variation in tract volume, comparing the aforementioned three versions of the 3T-DTI protocol to the 7T-DTI one, showed that despite resampling the 3T-DTI to having the equal number of directions and field-of-view, 7T-DTI still shows higher variation in volume than 3T-DTI in most of the tracts (Figure 16 in Appendix). Therefore, B1 inhomogeneity should be the primary cause of the increased volume variation.

A benefit of 7T MRI is that it has a higher baseline SNR than 3T MRI. Here, we might have undermined the sensitivity of our 7T-DTI protocol by acquiring images with the same resolution as in the 3T-DTI protocol (2.0 *mm* isotropic) rather than utilizing the higher baseline SNR for a higher resolution. Of note, De Santis et al. compared 3T-DTI and 7T-DTI at a higher image resolution than ours (1.5 *mm* isotropic) and found slightly higher effect sizes at 7T compared to 3T (31). One future direction could be the fusion of 3T and 7T, exploiting the perks of both worlds, with the high angular and spatial resolution, respectively (97). Apart from these image-protocol-related topics (refer to Table 4), there may also be microstructure-related differences between dMRI at 3T and 7T, as relaxation times may change by different amounts with a field strength in different compartments. Interestingly, the highest effect sizes in MD and AD were found with the 7T-DTI protocol. This might indicate that 3T and 7T are sensitive to different aspects of the pathophysiology in SLE. Overall, the lack of a clear advantage with using UHF dMRI in our study agrees with the main message of a recent review in which the author states that diffusion imaging at UHF, though still a worthwhile pursuit, has manifold associated challenges and converting the potential of higher field strengths into "better" diffusion imaging is by no means a straightforward task (106). More study is needed on a 7T-DTI protocol that leverages its benefits (higher SNR) and addresses its weaknesses [e.g., enhancement of B1+ homogeneity using parallel transmit (pTx) RF coils and RF pulse design approaches (107)].

**Table 4:** Overview of studies comparing 3T-DTI to 7T-DTI.

**References**	**Pathology**	**7T resolution in mm**	**Processing pipeline**	**Analysis method**	**Sensitivity metric**	**Most sensitive**	**Comment**
Sotiropoulos et al. (97)	HCP	1.05 x 1.05 x 1.05	Eddy and correction for gradient non-linearities	Voxel-wise deconvolution to assess crossing-fibers	Percentage of voxels with at least 2 crossing-fibers	None	Suggestion for a fusion, exploiting higher angular contrast of 3T-DTI and higher spatial resolution of 7T-DTI. Of note: spatial resolution at 7T was higher than at 3T (1.05 mm isotropic compared to 1.25 mm isotropic)
De Santis et al. (31)	MS	1.5 x 1.5 x 1.5	Eddy	TBSS	ANOVA	7T	3T-DTI and 7T-DTI were acquired at 1.5mm istotropic resolution

*HCP, Human Connectome Project; Eddy, eddy-current and motion correction; MS, Multiple Sclerosis; TBSS, Tract-based Spatial Statistics; ANOVA, ANOVA statistical package*.

In the analysis of processing pipelines, we noticed three consistent patterns of interest. The first was the minuscule differences across the data processing pipelines. Correcting for distortions from motion and eddy currents (Eddy) was the most beneficial, whereas gross smoothing reduced effect sizes by up to 20%. Interestingly, smoothing has been applied in many studies involving DTI or DKI (20, 39, 68, 80–84). In Maximov et al. (20), it was proposed that the pipeline most sensitive to pathology in terms of aging combines corrections for motion and eddy-current induced distortions, susceptibility deformations, denoising, bias field and Gibbs-ringing removal, together with field mapping and spatial smoothing. However, that study did not examine each step individually, but only cumulatively. In contrast to this, our study suggests that the choice of the processing pipeline does not play a crucial role, although smoothing should be avoided and motion and eddy current correction should be included. The latter is in line with our previous results, which showed that a motion and eddy current correction method capable of dealing with high *b*-value data reveals significant differences where a simpler and worse one did not (108). Note that this is the first time the study-level impact of individual state-of-the-art processing steps in dMRI has been analyzed.

We identified four primary strengths of the current study. The first strength is the relatively large sample sizes (31 HC and 77 patients with SLE). Second, all processing steps were evaluated independently. Third, the segmentation of the tracts was performed by an automatic pre-trained method, which allowed us to not only investigate tracts over the whole brain but also eliminate bias from subjective tracking. Fourth, the statistical analysis of the tracts took place in the native space of each subject instead of a template space. Previous studies have predominantly used the Tract-Based Spatial Statistics (TBSS) pipeline to perform voxel-wise statistical between-group comparison of DTI/DKI metrics on MNI152 space (109). However, by deriving WM skeletons from segmentations computed by thresholding FA maps, the TBSS approach lacks the ability to distinguish certain adjacent WM tracts, such as the inferior longitudinal and inferior fronto-occipital fasciculi and, thus, has limited capacity for anatomical specificity (110). Moreover, TBSS requires an accurate non-linear coregistration of the FA maps onto the MNI152 standard space and is, therefore, prone to misregistration errors that bias the final outcome of the study (111).

The study also has few limitations, and here, we consider four of them. First, we only examined one disease paradigm (SLE). Even though patients with SLE manifest a variety of neuropsychiatric symptoms that resemble many other neurodegenerative diseases (112, 113), the question is which of our results generalize to other diseases. Our results comparing DTI and DKI do not seem to generalize across diseases (Table 3), but we do expect that other pathologies will also exhibit large variations in effect sizes due to protocol, and this may be important to consider in meta-analyses. Furthermore, we do expect that our results concerning different pipelines will generalize, as well as our observation of reduced effect sizes from smoothing. Second, the pipelines we considered included neither outlier detection (52) nor harmonization (114). Regarding the former, however, the manual inspection did not reveal any clear outliers in our data, while harmonization was not necessary for our study, since we ran separate analyses on the different protocols and investigated between-group effect sizes per protocol: Cohen's *d* is a metric of groupwise differences in the mean normalized by the joint SD. When computed per protocol, inter-protocol biases do not need to be considered. Third, the resolution of DKI was lower than that of DTI (2.3 *mm* isotropic vs. 2.0 *mm* isotropic). As mentioned above, the poorer resolution might explain the lower effect sizes with DKI, in particular, given that smoothing led to reduced effect sizes. Fourth, not all subjects were scanned with all three protocols, which might have induced some systematic sample-related differences in the results. However, the majority of subjects overlapped and the overall picture did not change when including only matching subjects (refer to Supplementary Material).

## 5. Conclusion

In conclusion, effect sizes for detecting WM changes in patients with SLE were higher for DTI than DKI and higher for 3T than 7T. However, our results suggest that adjustments could be made to improve the protocols. For example, the sensitivity of 7T-DTI could potentially be enhanced by leveraging the higher baseline SNR of 7T for higher image resolution. Similarly, high-performance gradient coils could be utilized to reduce echo times and, thereby support a higher image resolution in DKI. Among the processing choices, eddy current and motion correction increase effect sizes, while no clear benefits seemed from denoising (MPPCA) and Gibbs-ringing removal. Smoothing was clearly detrimental for the effect sizes. However, the choice of diffusion protocol had a much greater impact than the choice of processing strategy.

## Data Availability Statement

Data are available upon request to the authors. A formal data sharing agreement is required prior to sharing data. Raw imaging data cannot be shared due to legal concerns.

## Ethics Statement

The studies involving human participants were reviewed and approved by the Regional Ethical Review Board in Lund, Sweden. The patients/participants provided their written informed consent to participate in this study.

## Author Contributions

EK contributed to the development of the pipelines, the conception and design of the analysis, the interpretation of the results, analysis of data and drafted the work. SW contributed to the design of the pipelines, the interpretation of data for the work and the revision of the work for important intellectual content. TR contributed to the acquisition of data for the work and the revision of the work for important intellectual content. AW contributed to the development of the pipelines. LK contributed to the revision of the work for important intellectual content. MC supervised the design of the pipelines and contributed to the interpretation of data for the work and the revision of the work for important intellectual content. PS contributed to the acquisition of data for the work, the interpretation of data for the work and the revision of the work for important intellectual content. MN contributed to the conception and design of the analysis, the interpretation of data for the work and the revision of the work for important intellectual content. All authors contributed to the article and approved the submitted version.

## Funding

The study was supported by Regional Research Funds (RegSkane-824651) (PS), SUS Foundation and Donations funds (PS), Alfred Österlund Foundation (PS), Swedish Rheumatism Association R-568371 (PS), and King Gustaf V's 80-year Foundation (FAI-2019-0559) (PS).

## Conflict of Interest

The authors declare that the research was conducted in the absence of any commercial or financial relationships that could be construed as a potential conflict of interest.

## Publisher's Note

All claims expressed in this article are solely those of the authors and do not necessarily represent those of their affiliated organizations, or those of the publisher, the editors and the reviewers. Any product that may be evaluated in this article, or claim that may be made by its manufacturer, is not guaranteed or endorsed by the publisher.
