# Supplementary material for: Sensitivity of Diffusion MRI to White Matter Pathology: Influence of Diffusion Protocol, Magnetic Field Strength, and Processing Pipeline in Systemic Lupus Erythematosus
Source: Front Neurol. 2022 Apr 26;13:837385. doi: 10.3389/fneur.2022.837385 (PMC9087851; doi:10.3389/fneur.2022.837385)
Supplement: Supplementary file 1 [file Data_Sheet_1.pdf]

Sensitivity of diffusion MRI to white matter pathology: influence of diffusion protocol, magnetic field strength and processing strategy in systemic lupus erythematosus

Evgenios N. Kornaropoulos *, **Stefan Winzeck, Theodor Rumetshofer, Anna Wikstrom, Linda Knutsson, Marta M. Correia, Pia C. Sundgren and Markus Nilsson**

Correspondence*:
Evgenios N. Kornaropoulos
evgenios.kornaropoulos@med.lu.se

APPENDIX

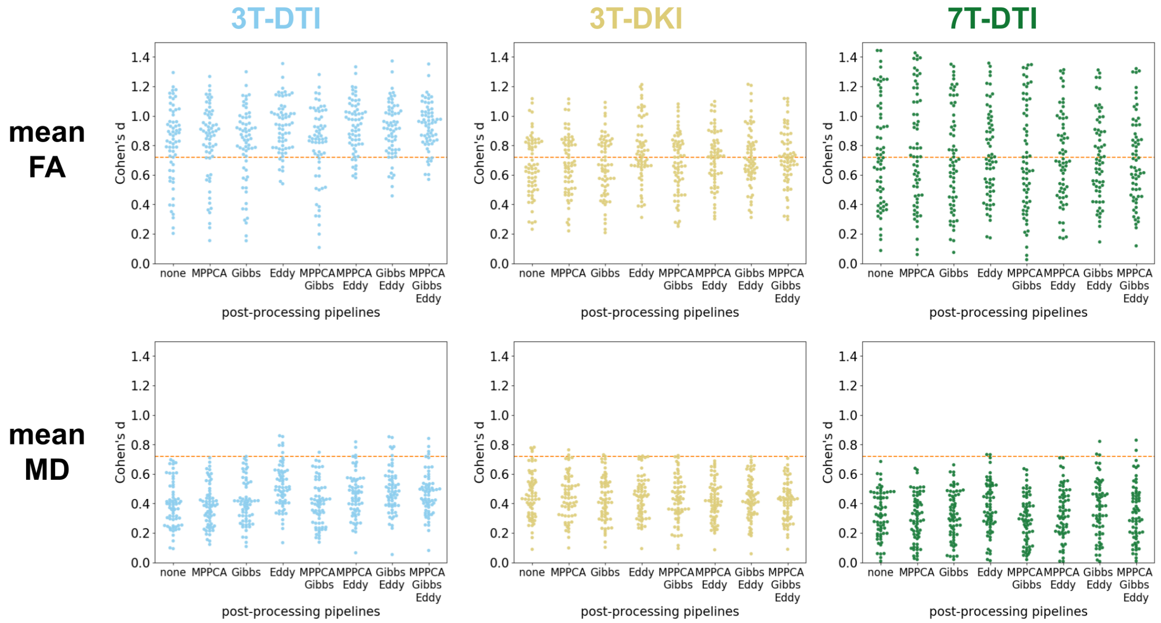

Figure 11: Impact of processing pipeline. The difference with Figure 5 is that the results in the current figure have been derived by using only the subjects that were scanned with all three acquisition protocols. The impact of the processing pipelines (x-axis) is evaluated through effect size estimates (Cohen's d scores, y-axis). Top row: effect size estimates in mean FA from 3T-DTI (top left), 3T-DKI (top center) and 7T-DTI (top right). Bottom row: effect size estimates with mean MD from 3T-DTI (top left), 3T-DKI (top center) and 7T-DTI (top right). No smoothing was applied. Each dot represents one tract. An orange dotted line in each plots defines the threshold in effect size above which the result is considered significant.

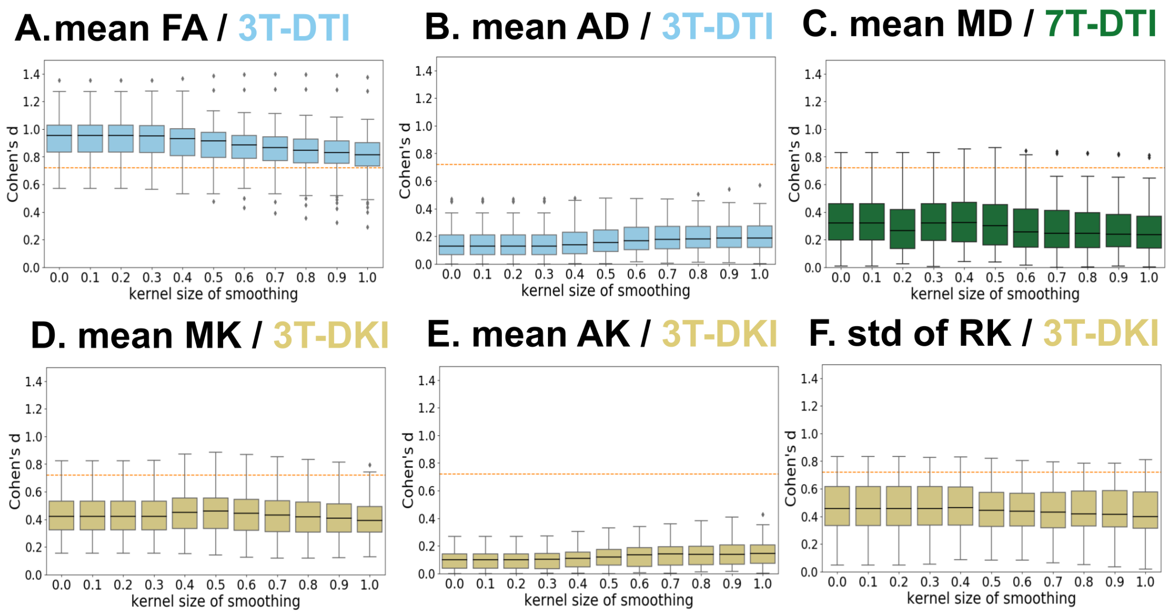

Figure 12: Influence of Gaussian smoothing. The difference with Figure 7 is that the results in the current figure have been derived by using only the subjects that were scanned with all three acquisition protocols. Different levels of Gaussian smoothing (sigma, x-axis) were evaluated by the effect size estimates (Cohen's d, y-axis). Panels A-F shows the effect on mean FA from 3T-DTI, mean AD from 3T-DTI, mean MD from 7T-DTI, mean MK from 3T-DKI, mean AK from 3T-DKI, and standard deviation of RK from 3T-DKI. The box plots show Cohen's d scores among tracts. Overall, smoothing had a detrimental influence on effect sizes. An orange dotted line in each plots defines the threshold in effect size above which the result is considered significant.

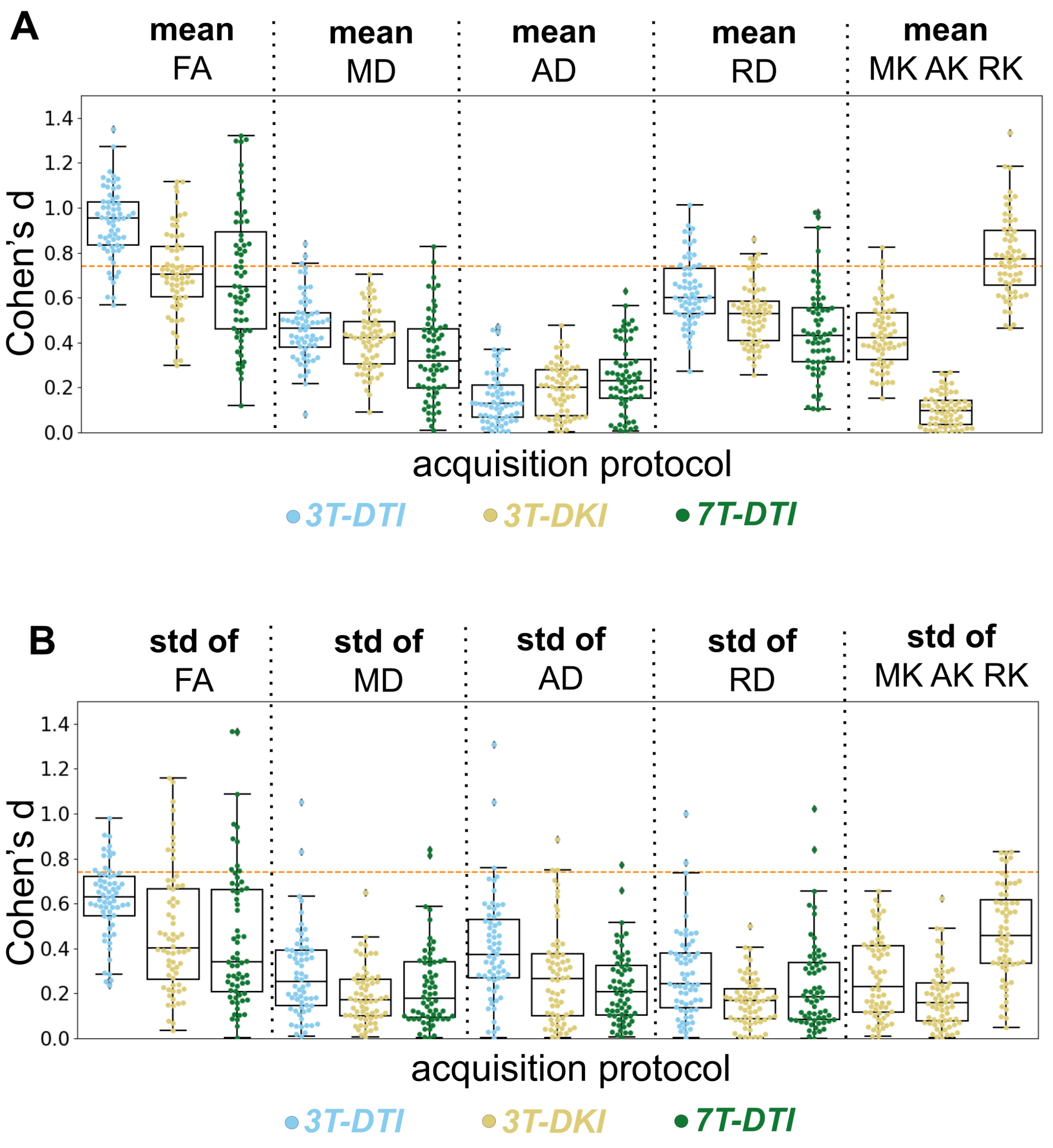

Figure 13: Evaluation of the acquisition protocol on the effect size based on subjects that were scanned with all three acquisition protocols. Columns show different diffusion parameters obtained with different protocols 3T-DTI (cyan), 3T-DKI (gold) or 7T-DTI (green). The mean (panel A) and standard deviation (panel B) of each diffusion parameter, derived for each protocol were examined. Pipeline VII was used in all cases. No smoothing was applied. The swarm and box plots show the distribution and quartiles (together with the median) of Cohen's d values among tracts respectively. Overall, 3T-DTI was the acquisition protocol that yielded the three most sensitive diffusion parameters (mean FA, standard deviation of FA and mean RD), and exhibited effect sizes above 0.54 (a threshold of significance before correcting for multiple comparisons) in the largest number of tracts. Moreover, the results between 3T-DTI and 7T-DTI were more similar than those between 3T-DTI and 3T-DKI, which implies that the choice of the diffusion protocol impacted the effect size analysis more than the choice of the magnetic field strength. Kurtosis parameters did not yield significant effect sizes, with the exception of the standard deviation of RK, that had significant effects in a small number of tracts. An orange dotted line in each plots defines the threshold in effect size above which the result is considered significant.

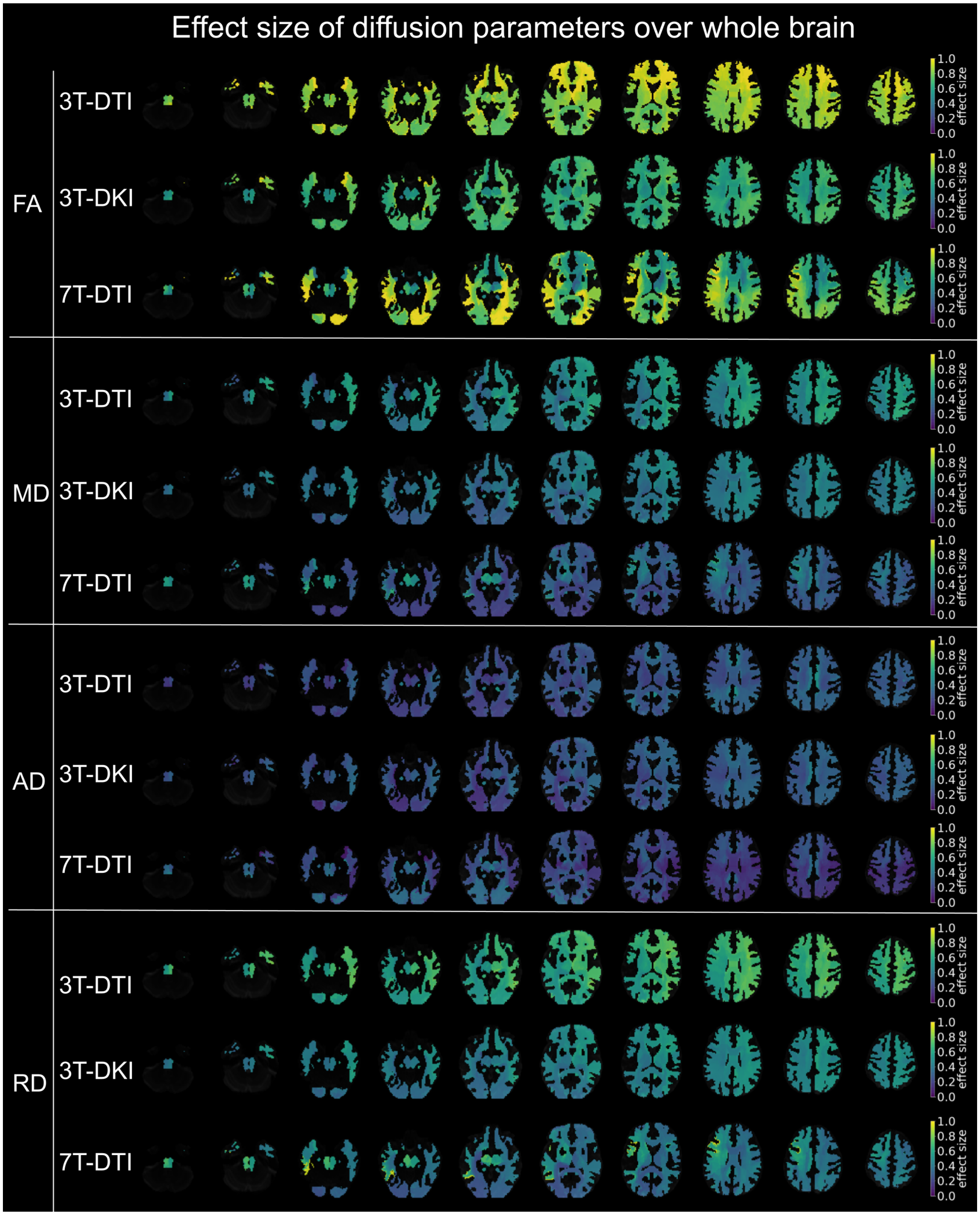

Figure 14: Effect size of diffusion tensor parameters over the whole brain, when derived by the different acquisition protocols and based on subjects that were scanned with all three acquisition protocols. Effect sizes in the depicted brains range from 0.0 (deep purple areas) to 1.0 (yellow areas). Overall, FA and RD were the parameters with the highest effect sizes across all three acquisition protocols, but the FA derived by 3T-DTI was the diffusion indice with highest number of areas in the brain exceeding the value 0.54 of an effect size. In the case of FA, 3T-DTI (top panel, first row) yielded the highest number of tracts with significantly large effect size than 3T-DKI (top panel, second row) and 7T-DTI (top panel, third row) since the brains in the top panel appear brighter than the brains in the second and third panels.

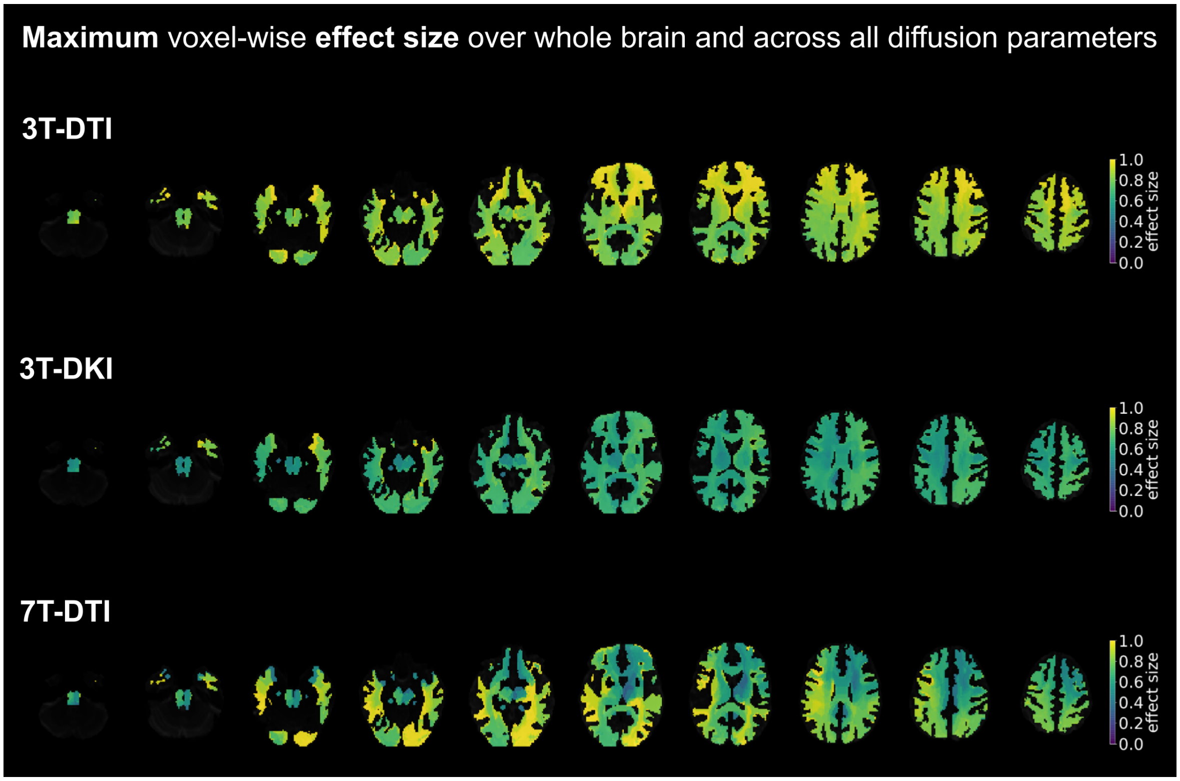

Figure 15: A summary of the spatial distribution of effect sizes over the whole brain, taking the maximum voxel-wise effect sizes across all diffusion tensor and kurtosis parameters (FA, MD, AD and RD for all three acquisition protocols plus MK, AK and RK in case of 3T-DKI) and including only those subjects that were scanned with all three acquisition protocols. Effect sizes in the depicted brains range from 0.0 (deep purple areas) to 1.0 (yellow areas). Overall, 3T-DTI was the protocol with the highest effect sizes among all three acquisition protocols, with more voxels with an effect size exceeding the value 0.54, which was the threshold for significance (turquoise colors).

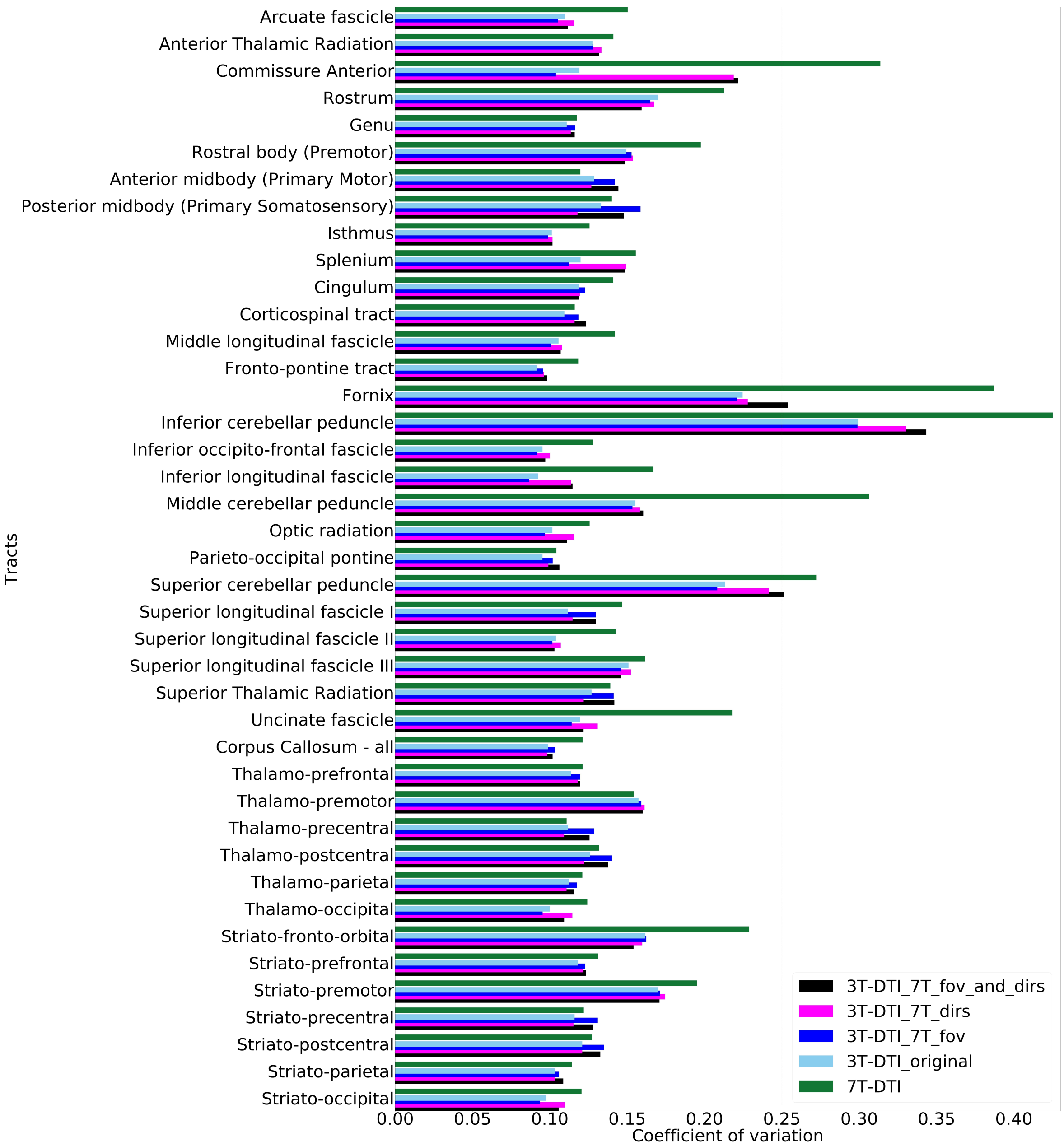

Figure 16: Evaluation of volume variation in the tracts, only for comparing 3T-DTI to 7T-DTI. The tract segmentation was based on data acquired with the original 3T-DTI (cyan), an alternative 3T-DTI in which the FOV was the same as in 7T-DTI (blue), a second alternative 3T-DTI in which the number of directions was equal to the number of directions in 7T-DTI (magenta), a thrid alternative 3T-DTI in which both the FOV was the same as and the number of directions equal to 7T-DTI (black) and finally the original 7T-DTI (green) acquisition protocol. In cases where TractSeg provides the left and right part of one fiber-tract as two different tracts (e.g. in case of left and right arcuate fasciculus), the left and right parts of the tract were averaged. The vertical dashed line shows the threshold of 0.25, which corresponds to a high variation.

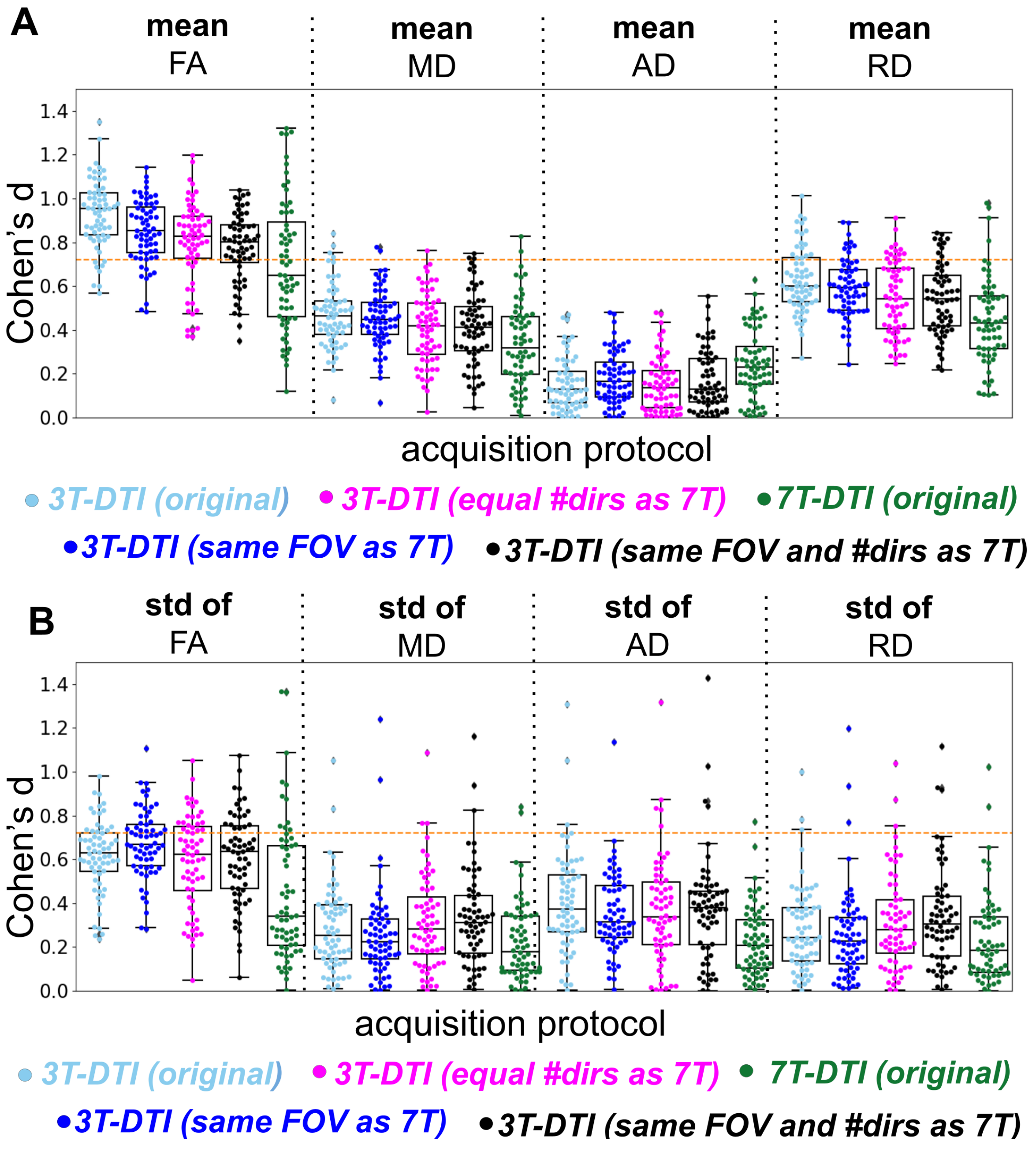

Figure 17: Evaluation of the effect the magnetic field strength (3T or 7T) had on the effect size. Columns show different diffusion parameters obtained with different protocols: the original 3T-DTI (cyan), an alternative 3T-DTI in which the FOV was the same as in 7T-DTI (blue), a second alternative 3T-DTI in which the number of directions was equal to the number of directions in 7T-DTI (magenta), a thrid alternative 3T-DTI in which both the FOV was the same as and the number of directions equal to 7T-DTI (black) and finally the original 7T-DTI (green). The mean (panel A) and standard deviation (panel B) of each diffusion parameter, derived for each protocol were examined. Pipeline VII was used in all cases. No smoothing was applied. The swarm and box plots show the distribution and quartiles (together with the median) of Cohen's d values among tracts respectively. Overall, 3T-DTI yielded higher effect sizes than 7T-DTI, even in the case where both the FOV and the number of directions were adopted to match the corresponding ones with 7T-DTI.
